# Metabolic Strategies Shared by Basement Residents of the Lost City Hydrothermal Field

**DOI:** 10.1128/aem.00929-22

**Published:** 2022-08-11

**Authors:** William J. Brazelton, Julia M. McGonigle, Shahrzad Motamedi, H. Lizethe Pendleton, Katrina I. Twing, Briggs C. Miller, William J. Lowe, Alessandrina M. Hoffman, Cecilia A. Prator, Grayson L. Chadwick, Rika E. Anderson, Elaina Thomas, David A. Butterfield, Karmina A. Aquino, Gretchen L. Früh-Green, Matthew O. Schrenk, Susan Q. Lang

**Affiliations:** a School of Biological Sciences, University of Utah, Salt Lake City, Utah, USA; b Bigelow Laboratory for Ocean Sciences, East Boothbay, Maine, USA; c Department of Molecular and Cell Biology, University of California, Berkeley, California, USA; d Department of Biology, Carleton College, Northfield, Minnesota, USA; e Joint Institute for the Study of Atmosphere and Ocean, University of Washington, Seattle, Washington, USA; f Department of Earth Sciences, ETH Zurich, Zurich, Switzerland; g Department of Earth and Environmental Sciences, Michigan State University, East Lansing, Michigan, USA; h School of the Earth, Ocean, and Environment, University of South Carolina, Columbia, South Carolina, USA; Kyoto University

**Keywords:** acetogenesis, formate, hydrogenase, hydrothermal, metagenomics, methanogenesis, serpentinization, sulfate reduction

## Abstract

Alkaline fluids venting from chimneys of the Lost City hydrothermal field flow from a potentially vast microbial habitat within the seafloor where energy and organic molecules are released by chemical reactions within rocks uplifted from Earth’s mantle. In this study, we investigated hydrothermal fluids venting from Lost City chimneys as windows into subseafloor environments where the products of geochemical reactions, such as molecular hydrogen (H_2_), formate, and methane, may be the only available sources of energy for biological activity. Our deep sequencing of metagenomes and metatranscriptomes from these hydrothermal fluids revealed a few key species of archaea and bacteria that are likely to play critical roles in the subseafloor microbial ecosystem. We identified a population of *Thermodesulfovibrionales* (belonging to phylum *Nitrospirota*) as a prevalent sulfate-reducing bacterium that may be responsible for much of the consumption of H_2_ and sulfate in Lost City fluids. Metagenome-assembled genomes (MAGs) classified as *Methanosarcinaceae* and Candidatus Bipolaricaulota were also recovered from venting fluids and represent potential methanogenic and acetogenic members of the subseafloor ecosystem. These genomes share novel hydrogenases and formate dehydrogenase-like sequences that may be unique to hydrothermal environments where H_2_ and formate are much more abundant than carbon dioxide. The results of this study include multiple examples of metabolic strategies that appear to be advantageous in hydrothermal and subsurface alkaline environments where energy and carbon are provided by geochemical reactions.

**IMPORTANCE** The Lost City hydrothermal field is an iconic example of a microbial ecosystem fueled by energy and carbon from Earth’s mantle. Uplift of mantle rocks into the seafloor can trigger a process known as serpentinization that releases molecular hydrogen (H_2_) and creates unusual environmental conditions where simple organic carbon molecules are more stable than dissolved inorganic carbon. This study provides an initial glimpse into the kinds of microbes that live deep within the seafloor where serpentinization takes place, by sampling hydrothermal fluids exiting from the Lost City chimneys. The metabolic strategies that these microbes appear to be using are also shared by microbes that inhabit other sites of serpentinization, including continental subsurface environments and natural springs. Therefore, the results of this study contribute to a broader, interdisciplinary effort to understand the general principles and mechanisms by which serpentinization-associated processes can support life on Earth and perhaps other worlds.

## INTRODUCTION

The fixation of carbon dioxide into organic carbon by autotrophic organisms is the foundation of all ecosystems on Earth. Even in subsurface environments, organic carbon is provided by fixation of carbon dioxide by chemoautotrophs or from the degradation of organic carbon originally produced in photosynthetic ecosystems and transported into the subsurface. However, organic carbon can form abiotically in hydrothermal environments, particularly in those that favor a set of geochemical reactions collectively known as serpentinization (1, 2). Microbial communities in serpentinizing environments are likely to benefit from the abiotic synthesis of simple organic compounds, but the processes and mechanisms that may allow this to occur have only recently been studied (3–5).

The Lost City hydrothermal field is located near the summit of the Atlantis Massif, a submarine mountain formed by the uplift of ultramafic rocks from Earth’s upper mantle and emplacement onto the seafloor along a major fault zone (6–8). Serpentinization of the Atlantis Massif results in the generation of H_2_ and hydrothermal fluids that are rich in formate, methane, and perhaps other forms of organic carbon (9–11). Dissolved inorganic carbon is vanishingly rare in the pH 9 to 11 hydrothermal fluids that vent from Lost City chimneys because it is either reduced to formate or methane, or is precipitated as carbonate minerals (11, 12). Sulfate, in contrast, appears to be an available oxidant throughout the subseafloor because it is never completely consumed by the relatively moderate hydrothermal conditions within the Atlantis Massif (4, 6).

Dense biofilm communities coating the surfaces of Lost City chimneys are capable of utilizing this bounty of energy and carbon released from the mantle (10, 13). However, these biofilms form in mixing zones where warm, anoxic hydrothermal fluids vent into cold, oxic seawater. These conditions may not be representative of subseafloor environments within the Atlantis Massif where habitats are probably confined to sparsely distributed fractures and channels within rocks that have limited exposure to seawater (8, 14). In particular, dissolved inorganic carbon is provided by ambient seawater to chimney biofilm communities, while its availability is severely limited in subseafloor habitats dominated by the products of serpentinization.

The microbiology of fluids venting from Lost City chimneys has been explored in only one study (15), as all other microbiological research at Lost City has focused on the chimney biofilms (4, 9, 10, 13, 16, 17). That early census of microbial diversity identified several novel 16S rRNA gene sequences, but they were poorly classified due to the limitations of microbial taxonomy at the time (15). In particular, the presence of potential sulfate-reducing bacteria (SRB) in Lost City fluids has been a mystery despite clear biogeochemical trends that indicate widespread SRB activity in the subseafloor (4, 10).

A deep-sea expedition to the Lost City in 2018 was designed to fill this knowledge gap by investigating the microbiology and biogeochemistry of fluids venting from Lost City chimneys (18). We exploited natural biogeochemical trends in fluids venting from distinct chimney locations within the Lost City field to test hypotheses about subseafloor microbial metabolic activity. Here, we report initial results from the sequencing of DNA and RNA in Lost City fluids, including the first sequences of metagenomes and metatranscriptomes from Lost City hydrothermal fluids. We identify a few key archaea and bacteria that appear to be indicative of subseafloor habitats strongly influenced by serpentinization. These results highlight metabolic strategies and adaptations that are common to life fueled by the products of serpentinization, including the potential use of formate and other simple forms of organic carbon as the primary sources of carbon for the ecosystem.

## RESULTS

### Characteristics of Lost City hydrothermal fluid samples.

**FIG 1:**
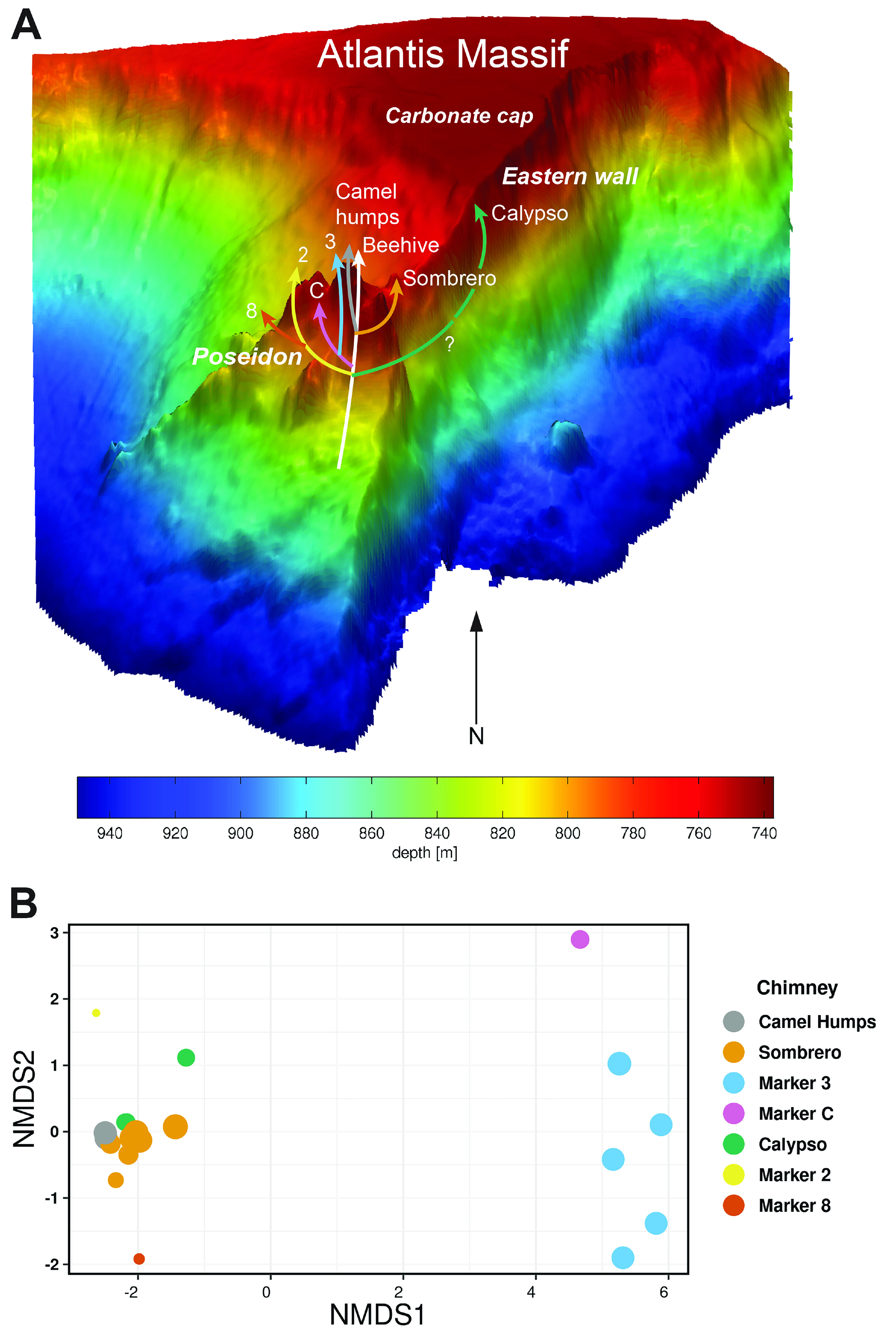
The Lost City hydrothermal field is located at 30°N, west of the Mid-Atlantic Ridge, on the southern wall of the Atlantis Massif. Part A shows a three-dimensional view of the field (after Kelley et al., 2005 [6]) featuring the massive Poseidon structure, which is composed of several actively venting chimneys. Hypothetical flow paths are informed by the aqueous geochemistry results reported here, by Aquino et al. (in review [40]), and by prior studies referenced in the main text. Part B is a nonmetric multidimensional scaling (NMDS) ordination of 16S rRNA amplicon sequence data where each data point represents the microbial community composition of one hydrothermal fluid sample. Sizes of data points are scaled to the measured sulfate concentration of that sample (Table 1).

Hydrothermal fluid samples were collected from actively venting chimneys at the Lost City hydrothermal field (Fig. 1; Fig. S1) using ROV *Jason* during the 2018 Lost City expedition aboard R/V *Atlantis* (AT42-01). This study includes 39 samples of hydrothermal fluids dedicated to DNA and RNA sequencing, including analyses of amplicon sequence variants (ASVs), metagenomes, and metatranscriptomes (Table 1; Table S1).

**TABLE 1:** Overview of hydrothermal fluid samples collected from Lost City chimneys

Chimney location	Metagenome libraries	Metatranscriptome libraries	16S rRNA amplicon libraries (DNA)	16S rRNA amplicon libraries (RNA)	Cells per mL^*a*^	Ma× T (°C)^*b*^	pH	Sulfide (mmol/kg)	Sulfate (mmol/kg)	Mg (mmol/kg)
Camel Humps	2	0	4	0	4.3 × 10^4^	41	8.1	0.02	24	47
Sombrero1	3	1	7	1	5.9 × 10^4^	13	8.0	0.002	27	54
Sombrero2	2	0	6	1	6.3 × 10^4^	52	8.7	0.12	18	36
Marker 3	2	0	6	0	6.3 × 10^4^	21	8.6	0.20	23	45
Marker C	0	0	1	1	3.8 × 10^4^	80	10.1	0.39	16	31
Calypso	2	0	6	1	4.8 × 10^4^	24	9.3	1.3	15	30
Marker 2	2	1	3	1	2.6 × 10^4^	58	9.5	1.8	8	15
Marker 8	0	0	1	0	7.5 × 10^4^	42	9.9	1.8	9	19

a Cell numbers are the median of all samples collected from that location.

b Temperatures and chemistry values are reported for one representative sample collected from that location, typically the sample for which the most chemistry and/or sequence data were available.

The fluid samples ranged from those that were barely distinguishable from ambient seawater (~11°C, pH 8) to warm and highly alkaline hydrothermal fluids (~80°C, pH 10). Direct counts of visible cells showed little variability among fluids, with densities approximately 2 to 8 × 10^4^ mL^−1^ in all samples, although the two samples with the highest temperatures had the least number of cells (Table 1).

**FIG 2:**
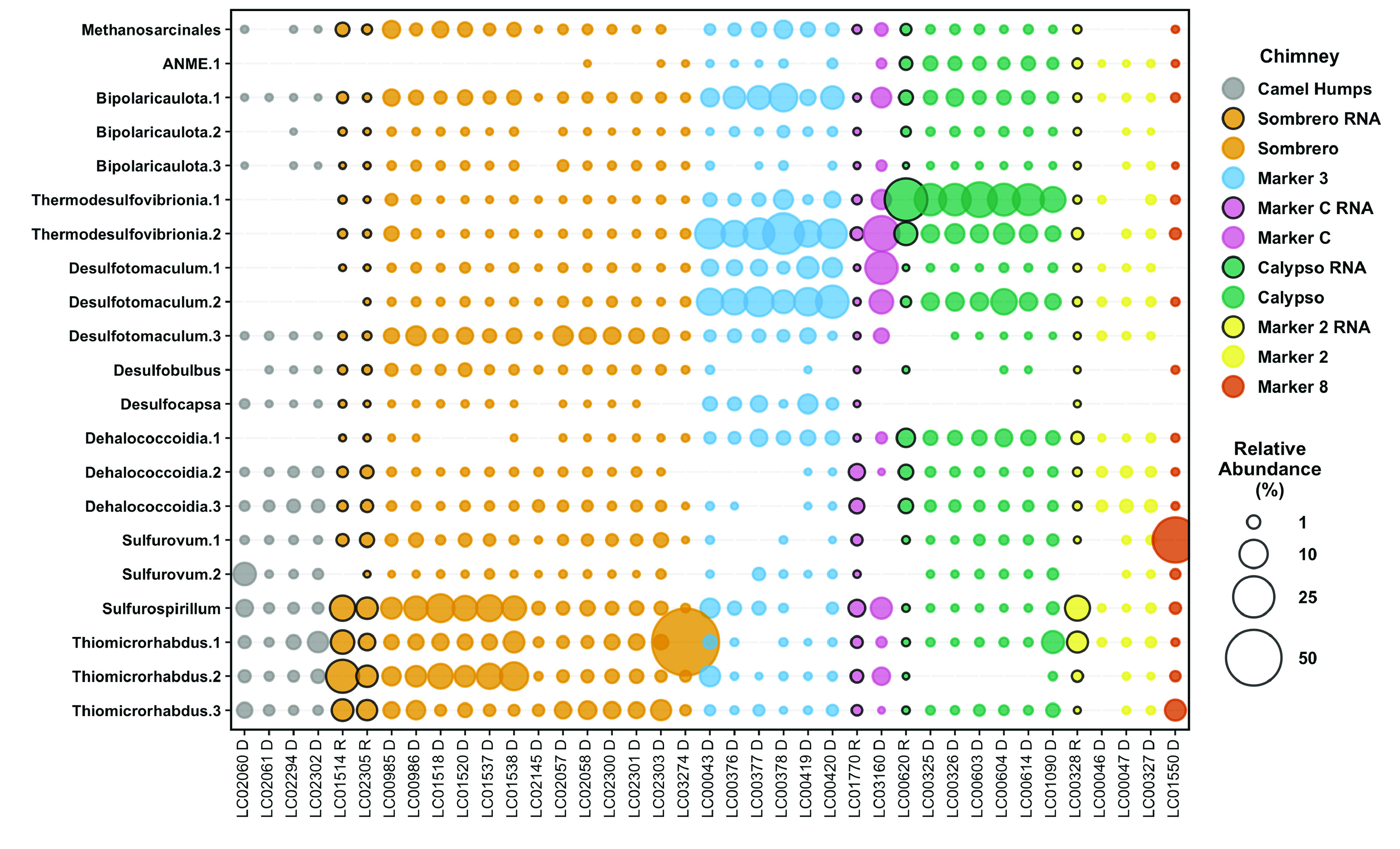
Relative abundances of selected ASVs in Lost City hydrothermal fluid samples. Amplicon libraries were generated from both DNA and RNA extractions; bubbles representing relative abundances in RNA libraries are highlighted with black borders. ASVs were selected to highlight the taxa that were the focus of this study, as well as additional taxa that are expected to be associated with hydrothermal environments and provide context for interpreting differences among fluid samples. A full table of ASV counts is provided in Table S2.

Fluids venting from markers 3 and C contained ASV compositions that were notably distinct from those of all other fluids (Fig. 1), including high relative abundances of *Thermodesulfovibrionia*, *Desulfotomaculum*, and *Bipolaricaulota* (Fig. 2; Table S2). In addition, marker 3 fluids were rich in metagenomic sequences classified as family *Methanosarcinaceae*, which includes the dominant archaeal phylotype previously detected in Lost City chimneys (16, 17, 19). The greater representation of archaeal sequences in the metagenomes suggests a bias against archaeal sequences in the ASV data set.

Fluids venting from Camel Humps were more diverse than fluids from markers 3 and C (~4,000 versus ~200 observed ASVs and Inverse Simpson index of approximately 100 to 200 versus 10 to 50; Table S2), and contained likely chimney-associated taxa such as *Sulfurovum*, *Sulfurospirillum*, and *Thiomicrorhabdus* (15, 16) at similar abundances as taxa typically associated with ambient seawater (e.g., *Alteromonas*, *Roseobacter*, *Halomonas*). The overall microbial community structure of Sombrero fluids is broadly similar to that of Camel Humps fluids, although warmer and more sulfidic Sombrero fluids included greater proportions of taxa that were also abundant in fluids from markers 3 and C (Fig. 2). Fluid samples from the chimneys at markers 2 and 8 contained a similar combination of chimney-associated and seawater-derived taxa, although marker 2 fluids were particularly dominated by *Alteromonas* and marker 8 fluids by *Sulfurovum* (Table S2).

In general, the proportion of ambient seawater in each hydrothermal fluid sample, as measured by Mg concentration, did not predict the presence of microbes likely to inhabit anoxic, subseafloor environments. Instead, the distribution of anaerobic taxa most likely to be strongly linked with serpentinization (e.g., *Methanosarcinaceae*, *Thermodesulfovibrionia*, *Desulfotomaculum*, and *Bipolaricaulota*) was strongly chimney-specific, indicating a strong influence of subsurface conditions that is only weakly mitigated by the mixture of seawater during sampling. Detailed comparisons of the hydrothermal fluid samples are provided in the supplemental material.

### Metagenome-assembled genomes.

**FIG 3:**
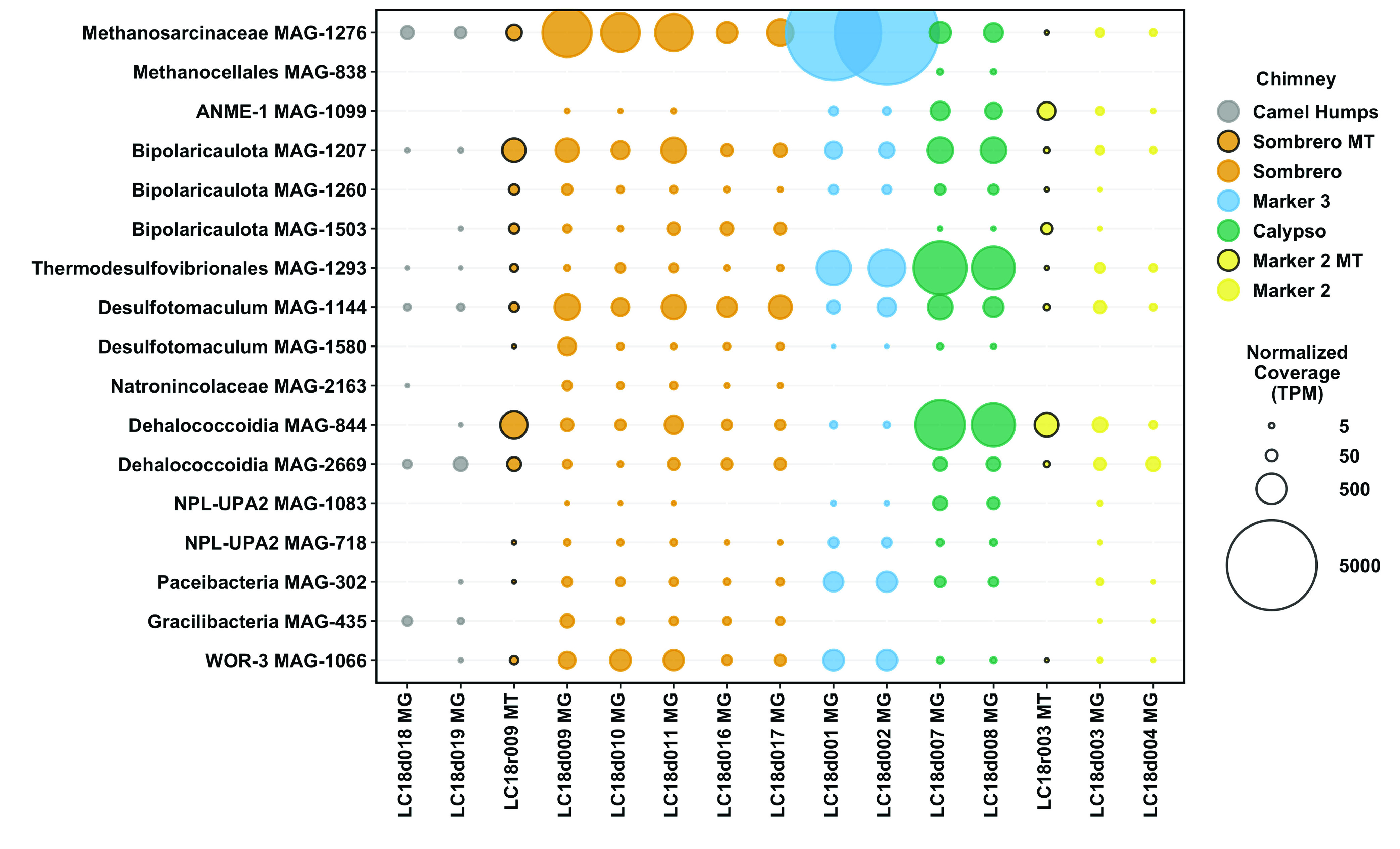
Abundance of refined MAGs in Lost City hydrothermal fluid samples. Total mapped read coverage was normalized to genome size and to the size of the metagenome or metatranscriptome library. The final normalized coverage is reported as a proportional unit (transcripts/fragments per million; TPM) suitable for cross-sample comparisons. Bubbles representing coverage in metatranscriptomes (MT), rather than metagenomes (MG), are highlighted with black borders. For clarity, not all MAGs are shown. A full coverage table is provided in Table S4.

A total of 305 metagenome-assembled genomes (MAGs) with at least 50% estimated completion were recovered from the pooled “all fluids” assembly and the six chimney-specific assemblies (Fig. S3; Table S4). MAGs that were representative of the taxa enriched in markers 3 and C, as well as MAGs that contained key genes associated with the metabolism of H_2_, sulfate, formate, and methane, were selected for additional analyses. Reassembly and manual refinement of these sequences (see supplemental material) resulted in 30 refined and curated MAGs (Fig. 3) that are at least medium-quality (>50% complete, <10% redundancy, as defined by reference 20). Generally, these MAGs are most abundant in marker 3, Calypso, or Sombrero, and are nearly absent in Camel Humps and marker 2. Unfortunately, metagenomic sequences could not be obtained from marker C or marker 8.

A single *Methanosarcinaceae* MAG that corresponds to the same *Methanosarcinaceae* phylotype previously identified as a dominant member of Lost City chimney biofilm communities (13, 16, 17, 19), was especially abundant in the fluids from marker 3 (Fig. 3; Fig. S5). Although this is the highest-coverage MAG recovered by this study, its maximum coverage of ~6,000 transcripts/fragments per million (TPM) still represents only 0.6% of total mapped reads. Therefore, the few curated MAGs reported here are not an accurate census of the natural microbial community, but they were selected as representatives of the key taxa detected by the 16S rRNA and metagenomic surveys. Many of the 30 refined MAGs share similarities with 16S rRNA and/or metagenomic sequences from other sites associated with serpentinization. In particular, similar *Methanosarcinaceae*, *Thermodesulfovibrionales*, and Bipolaricaulota sequences (Fig. S5 to 7) have been identified in chimneys of the Old City and Prony Bay (21, 22).

Below, we briefly describe key features of these MAGs that seem relevant to an initial exploration of the Lost City subseafloor ecosystem, focusing on genes associated with the metabolism of H_2_, formate, sulfur, and methane. Additional information about each MAG is reported in the supplemental material, including detailed descriptions of genomic content and predicted protein functions (Tables S5 and 6).

### Hydrogenases.

**FIG 4:**
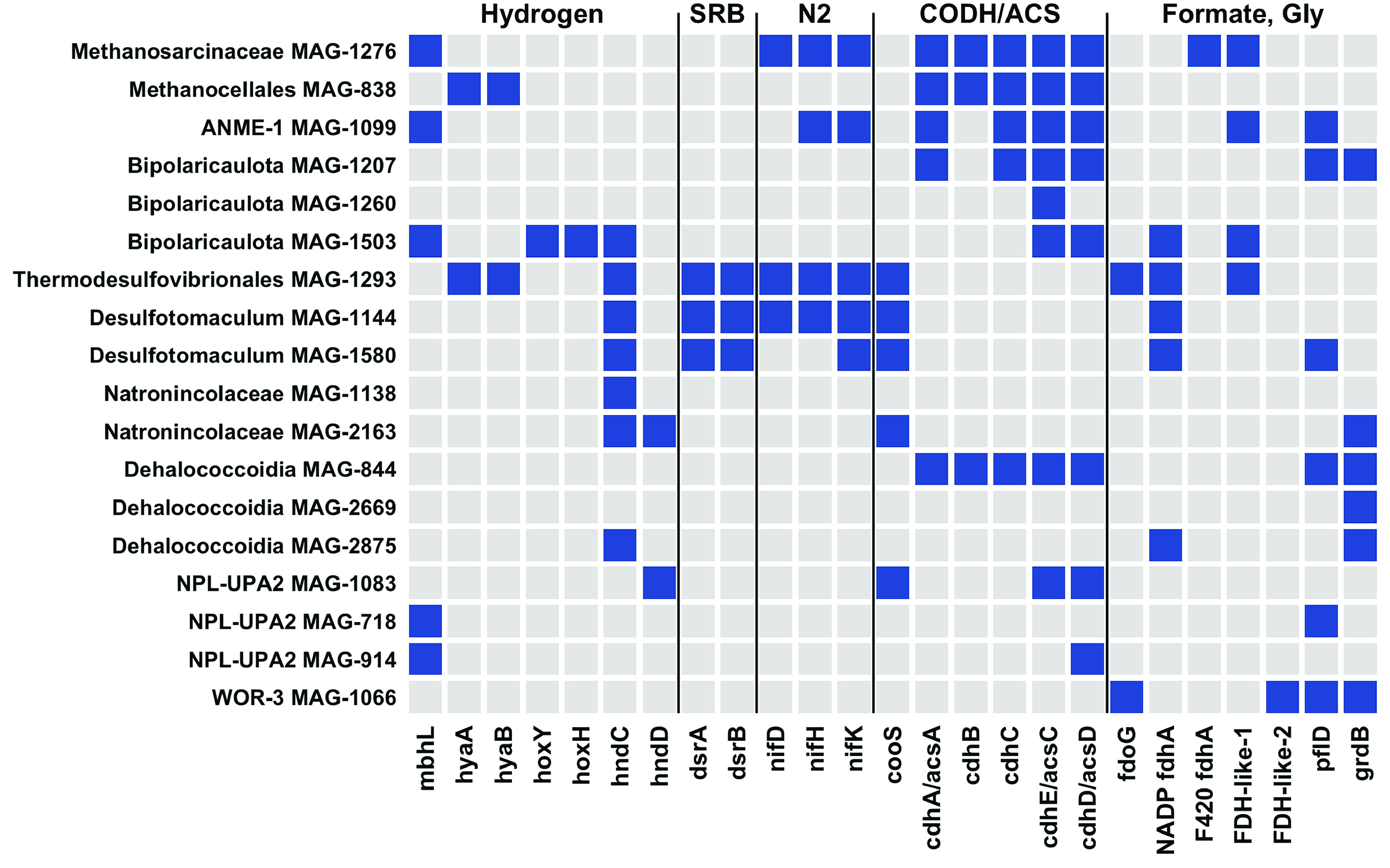
Presence and absence of key genes in refined MAGs. Genes defined by KEGG Orthology (see Table S5) were selected to highlight potential metabolic capabilities to metabolize hydrogen gas, to reduce sulfate to sulfide (SRB), to fix nitrogen (N_2_) gas, to fix carbon dioxide via the Wood-Ljungdahl pathway (CODH/ACS), and to utilize formate or glycine as carbon sources. Patescibacteria MAGs (including Paceibacteria and Gracilibacteria) are not shown here because they lack all of the gene shown here.

[NiFe]-hydrogenases were found in *Thermodesulfovibrionales* MAG-1293 (HyaAB), *Methanocellales* MAG-838 (HyaAB), and Bipolaricaulota MAG-1503 (HoxYH) (Fig. 4). Of these, the *Thermodesulfovibrionales* MAG was by far the most abundant in venting fluids (Fig. 3). *Methanosarcinaceae* MAG-1276 encodes two hydrogenases associated with methanogenesis: F_420_-reducing hydrogenase (FrhAB) and the small and large subunits of a second hydrogenase predicted to be EchCE by GhostKOALA and group 4e hydrogenase (ferredoxin-coupled, Ech-type) by HydDB. The two hydrogenase subunits are encoded on a contig that includes six subunits of a H^+^:Na^+^ antiporter. The MAG also encodes a formate dehydrogenase that can provide electrons to MvhD and HdrABC instead of the H_2_-oxidizing Vho/Vht enzyme (23). Thus, Lost City *Methanosarcinaceae* may power methanogenesis with electrons from both H_2_ and formate. The same MvhD-HdrABC complex, without FDH, was also found in MAGs classified as ANME-1, *Natronincolaceae*, and Bipolaricaulota (Table S5).

**FIG 5:**
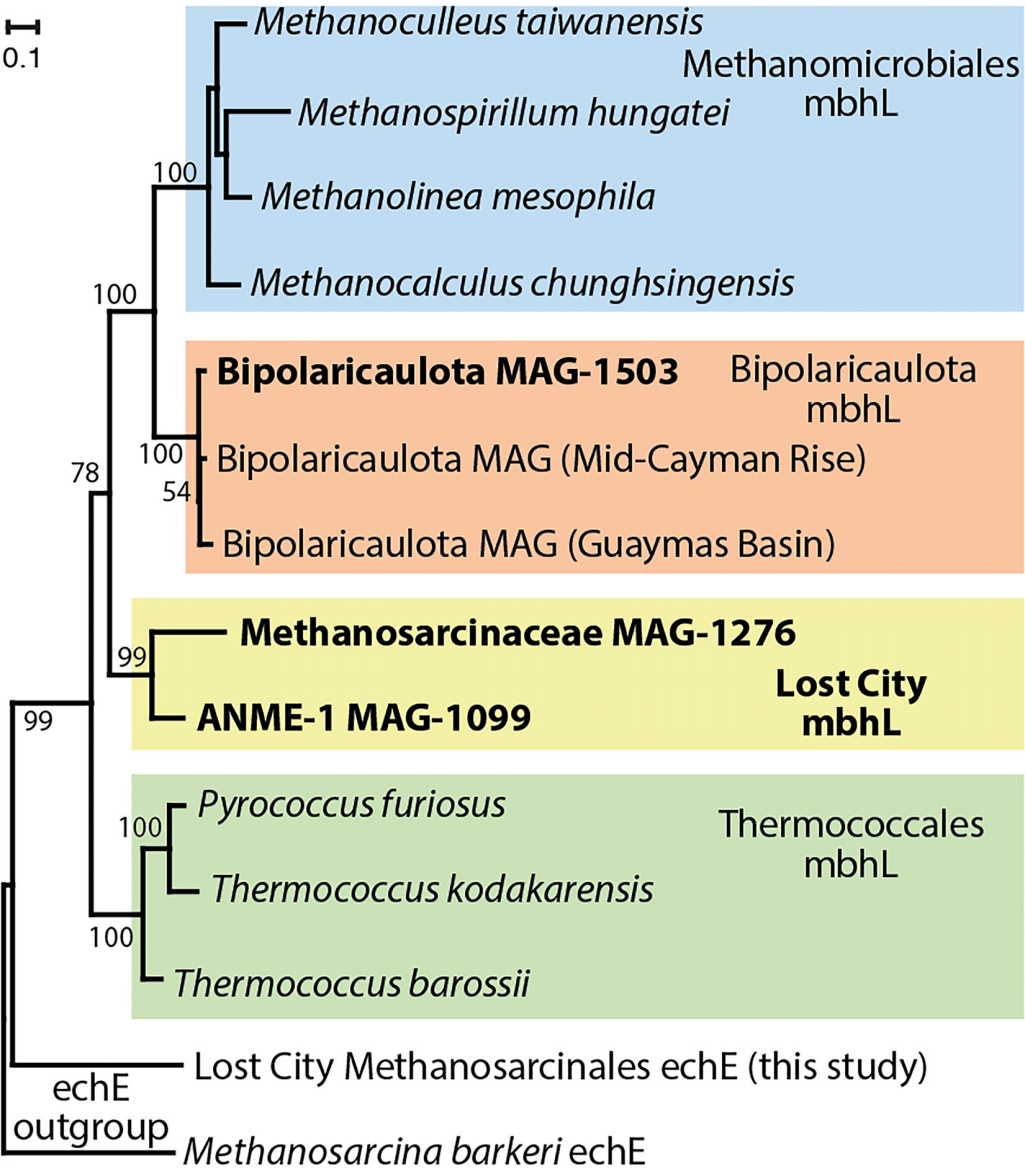
Phylogeny of the large catalytic subunit of membrane-bound hydrogenase (mbhL). Sequences identified in refined MAGs from this study are highlighted in bold font. The two archaeal sequences from Lost City (*Methanosarcinaceae* and ANME-1) form their own clade apart from all known mbhL sequences. The mbhL sequence from a Lost City Bipolaricaulota MAG clusters together with Bipolaricaulota MAGs from other hydrothermal environments. Bootstrap support values are shown for each node. An expanded version of this figure, including the gene order for the mbh gene cluster is provided as Fig. S8.

In addition, the *Methanosarcinaceae* and ANME-1 MAGs contain a complete 14-gene cluster (mbhA-N) encoding membrane-bound hydrogenase (Mbh) (Fig. 5; Fig. S8). For each predicted gene in the cluster, the homologs in the *Methanosarcinaceae* and ANME-1 MAGs are more similar to each other than to any other sequences in public databases. The same gene cluster, with conserved synteny, is also found in methanogens belonging to the order *Methanomicrobiales* and in heterotrophs of the order *Thermococcales* (24). The MbhL subunits from these methanogens have only 42% to 45% identities with the Lost City MbhL sequences reported here, which have greater similarly (~49% identities) to MbhL sequences from *Thermococcus*. Bipolaricaulota MAG-1503 also includes a predicted MbhL sequence, which is most closely related to two Bipolaricaulota MAGs from hydrothermal systems: the Mid-Cayman Rise (25) and Guaymas Basin (26) (Fig. 5).

[NiFe]-hydrogenase sequences (HyaAB) were also highly abundant in Sombrero and Camel Humps fluids (Table S7), where they were primarily encoded by *Thiomicrorhabdus*. We did not prioritize the analysis of *Thiomicrorhabdus* MAGs because our prior work indicated they inhabit oxygenated biofilm communities on chimney surfaces (27). We previously noted the absence of hydrogenase sequences phylogenetically linked with these bacteria (28), but recent sequencing of additional genomes from *Thiomicrospira*, *Thiomicrorhabdus*, and Hydrogenovibrio (29) has revealed many of the hydrogenase sequences in Lost City metagenomes are affiliated with these taxa after all.

[FeFe]-hydrogenases typically associated with the production of H_2_ during fermentation were represented by HndCD sequences in one *Natronincolaceae* and one NPL-UPA2 MAG (Fig. 4). This hydrogenase is also capable of H_2_ oxidation with reduction of NADP in some organisms (30). Therefore, the metabolic role of this hydrogenase in Lost City cannot be predicted with certainty.

### Formate dehydrogenase and transporters.

Formate dehydrogenase (FDH) catalyzes the reversible oxidation of formate to carbon dioxide, and various forms of FDH have diverse physiological roles in all three domains of life (31). Oxidation of formate was detected in shipboard incubations with ^13^C-labeled formate with all Lost City fluid samples, including those with significant contributions from ambient seawater (Table S8). However, none of the incubations supplemented with ^13^C-enriched formate or dissolved inorganic carbon (DIC) produced methane with a δ ^13^C that was distinguishable from that of methane native to Lost City fluids (approximately −10 ‰) (6, 11). Therefore, we conclude no methane was produced from DIC or formate during the seafloor incubations.

**FIG 6:**
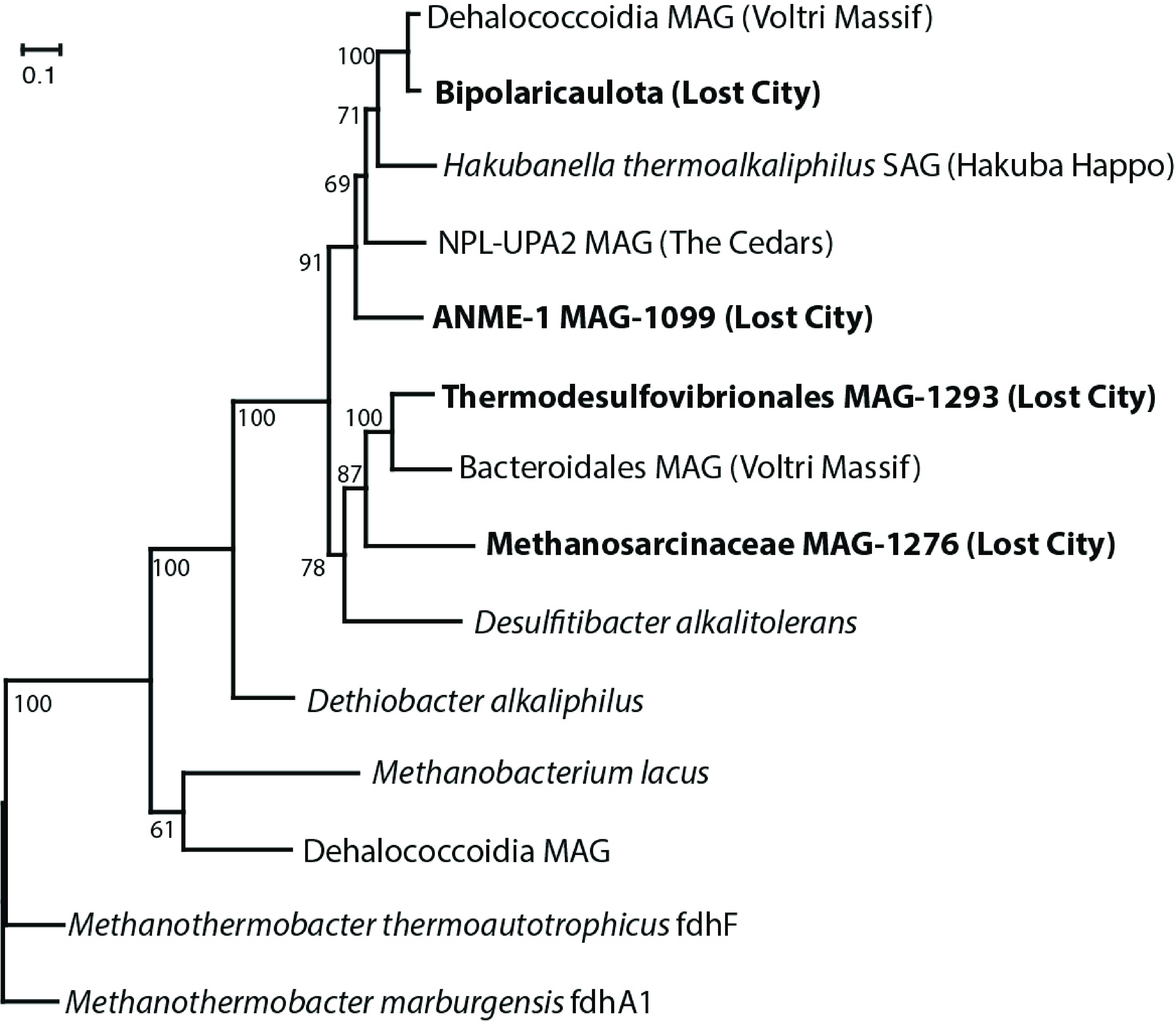
Phylogeny of divergent FDH-like sequences. Sequences identified in refined MAGs from this study are highlighted in bold font. Their closest matches in the NCBI nr database are from other serpentinite-hosted springs (Voltri Massif, Hakuba Happo, and The Cedars). The FDH-like sequences shown here include an iron-sulfur binding domain and a molybdopterin oxidoreductase domain, which are encoded as two separate coding regions in some species and as a fused coding region in others (see Fig. S9 an expanded version of this figure, including genomic context). The phylogeny was constructed from the conserved oxidoreductase domain. Bootstrap support values are shown for each node. The Lost City Bipolaricaulota sequence was identified in multiple BinSanity bins classified as Bipolaricaulota, but it was not included in the final, manually refined MAGs.

We identified at least three kinds of FDH in Lost City fluids plus two distinct variants of FDH-like sequences: (i) NAD(P)-dependent FDH catalyzing formate oxidation in bacteria (K00123; FdoG/FdhF/FdwA) was detected in *Thermodesulfovibrionales* and WOR-3 MAGs; (ii) NAD(P)-dependent FDH catalyzing reduction of carbon dioxide into formate (K05299; FdhA) was detected in Bipolaricaulota, *Thermodesulfovibrionales*, *Desulfotomaculum*, and *Dehalococcoidia* MAGs; (iii) F_420_-dependent FDH catalyzing formate oxidation in methanogens (K22516; FdhA) was detected in the *Methanosarcinaceae* MAG; (iv) a divergent FDH-like sequence was detected in *Methanosarcinaceae*, ANME-1, Bipolaricaulota, and *Thermodesulfovibrionales* MAGs; (v). another divergent FDH-like sequence, distinct from those described above such that it could not be reliably placed in the phylogeny of Fig. 6, was detected in the WOR-3 MAG (Fig. 4).

The divergent FDH-like sequence shared by the *Methanosarcinaceae*, ANME-1, Bipolaricaulota, and *Thermodesulfovibrionales* MAGs was identified by conducting blastp searches of the NCBI nr and JGI IMG databases for FDH homologs. Apparent homologs of these FDH-like sequences were also found in MAGs and SAGs (single-amplified genomes) from three continental serpentinite-hosted springs: the Voltri Massif in Italy (32), The Cedars in California, USA (33), and Hakuba Happo hot springs in Japan (34) (Fig. 6; Fig. S9). The serpentinization-associated FDH-like sequences share 65% to 94% amino acid identities with each other and approximately 40% to 50% amino acid identities with FDH sequences from characterized organisms, with the exception of Desulfitibacter alkalitolerans (63% amino acid identities). In a previous study, we reported these divergent FDH-like sequences as bacterial based on their similarity to *Desulfitibacter* sequences (10). Although Desulfitibacter alkalitolerans can use formate as an electron donor, it encodes two additional homologs of fdhA that are not present in any Lost City metagenomes. A similar FDH-like sequence is encoded by Dethiobacter alkaliphilus, which is unable to grow on formate as its sole carbon source (35). The phylogeny in Fig. 5 is rooted with FdhF (anaerobic formate hydrogen lyase) from Methanothermobacter thermautotrophicus and Methanothermobacter marburgensis. These two species are unable to grow on formate (36), and lack a separate fdhABC operon that is found in *M. thermautotrophicus* st. CaT2, which can grow on formate.

The formate transporters FdhC and FocA that were previously identified in Lost City chimney biofilms (10, 13) were also detected in the metagenomes of venting fluids reported here, but they were only present at very low coverage (Table S7). None of the MAGs highlighted by this study contain any known formate transporters. A lack of canonical formate transporters was also reported recently for a formate-utilizing methanogen in serpentinite-hosted, hyperalkaline groundwaters (37). Therefore, transport of formate into the cells of organisms inhabiting hyperalkaline subsurface environments may be carried out by uncharacterized proteins.

### Sulfate reduction.

**FIG 7:**
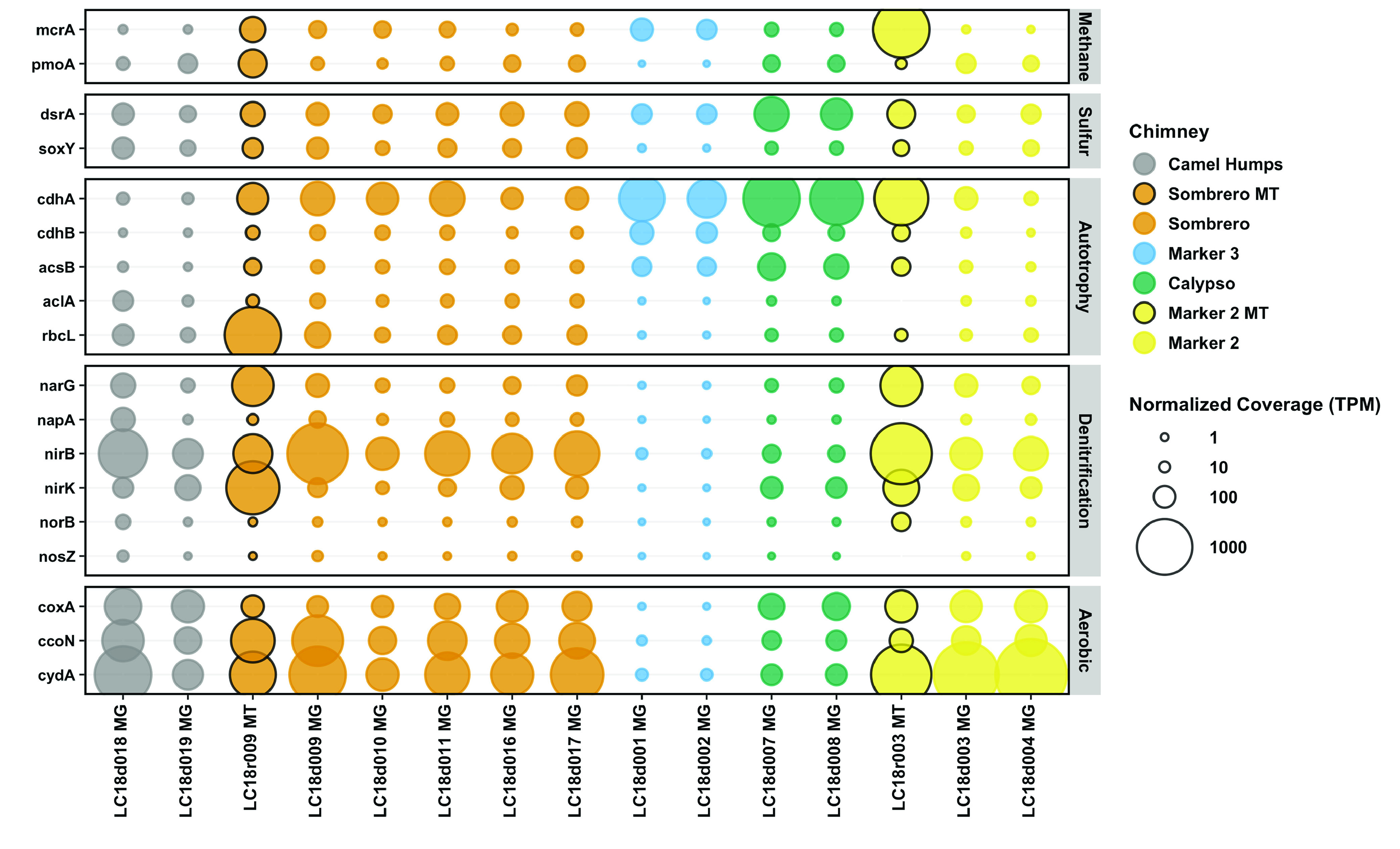
Abundance of key genes in Lost City hydrothermal fluid samples. Metagenomic coverage was normalized to predicted protein length and to the size of the metagenome or metatranscriptome library. The final normalized coverage is reported as a proportional unit (transcripts/fragments per million; TPM) suitable for cross-sample comparisons. Bubbles representing coverage in metatranscriptomes (MT), rather than metagenomes (MG), are highlighted with black borders. Genes are defined with KEGG Orthology; see Table S5.

Surprisingly, the samples of sulfidic fluids collected from the chimney at marker 2 (Table 1) did not contain elevated levels of taxa expected to represent SRB (Fig. 2 and 3) or the genes encoding dissimilatory sulfite reductase (DsrAB) (Fig. 7). Instead, marker 2 fluids are dominated by aerobic bacteria that are likely to be adapted to chimney biofilms or to shallow subsurface zones with exposure to ambient seawater. Potential SRB such as *Thermodesulfovibrionales* were most abundant in the fluids venting from marker 3, marker C, Sombrero, and Calypso (Fig. 2 and 3).

The other potential SRB in Lost City fluids include *Desulfotomaculum*, *Desulfocapsa*, and *Desulfobulbus. Desulfotomaculum* have been implicated as potential SRB in Lost City chimney biofilms (38), but sequences predicted to encode hydrogenases or carbon fixation enzymes were not identified in the *Desulfotomaculum* MAGs; therefore, their ability to reduce sulfate may depend on the availability of organic matter. Furthermore, some *Desulfotomaculum* species are known to be incapable of sulfate reduction despite encoding DsrAB (39). *Desulfobulbus* sequences were very rare in fluids from markers 3 and C. *Desulfocapsa* were moderately abundant in marker 3 fluids, but no MAGs classified as *Desulfocapsa* could be recovered during this study. Additionally, most of the dsrAB sequences in Lost City fluids were affiliated with *Thermodesulfovibrionales* or *Desulfotomaculum*; no dsrAB sequences belonging to *Desulfocapsa* or *Desulfobulbus* were identified in high-coverage contigs.

### Methane oxidation.

Methane is present in Lost City fluids at a remarkably constant concentration of ~1 mM, while concentrations of H_2_, sulfate, sulfide, and other chemicals vary widely (6, 9, 40). The source of the methane, i.e., whether it is synthesized abiotically as a product of serpentinization or released from carbon stored within basement rocks, remains uncertain (41–44). Oxidation of ^13^C-enriched methane to DIC with δ ^13^C values significantly greater than that of DIC native to Lost City fluids (<1.5 ‰) (6, 11) was detected in most Lost City fluid samples, except the sample of marker 3 fluids (Table S8).

The primary candidates for the anaerobic oxidation of methane at Lost City are the ANME-1 archaea, which are most abundant in Calypso fluids (Fig. 2 and 3). The Lost City ANME-1 MAG contains the core methanogenic pathway (Table S5), including F_420_-dependent methylenetetrahydromethanopterin reductase (Mer). This gene is required for methanogenesis from carbon dioxide, but it is typically absent in ANME genomes, with at least one exception previously reported (45). The MAG lacks all but one of the subunits of N^5^-methyl-H_4_MPT:coenzyme M methyltransferase (Mtr), which catalyzes the penultimate step of methanogenesis (and putatively the second step of reverse methanogenesis). It is present in most but not all ANME-1 genomes (46). The absence of cytochromes and presence of hydrogenases in the ANME-1 MAG was noted by Chadwick et al. (46) as consistent with the genomic features of the so-called “freshwater” clade of ANME-1, for which the genus “Candidatus Methanoalium” was proposed. One of the shared features within this clade, including the Lost City ANME-1 MAG, is a novel HdrABC-MvhADG complex (46), which is involved in the transfer of electrons derived from H_2_ to heterodisulfide and ferredoxin in methanogens. Therefore, this clade of ANME-1 may be involved in the H_2_-fueled production of methane instead of, or in addition to, the oxidation of methane. Distinguishing between methanogenesis and the anaerobic oxidation of methane with genomic data alone is notoriously difficult (46), and the *Methanosarcinaceae* and ANME-1 MAGs reported here contain features that are potentially consistent with both the production and oxidation of methane.

Potential methanotrophic bacteria were represented by ASVs classified as the gammaproteobacterial family *Methylomonaceae* (e.g., *Methylobacterium*), but they are expected to represent chimney biofilm communities (15) and were not abundant in any of the fluids included in this study. ASVs classified as *Methyloceanibacter*, various species of which can aerobically oxidize methane, methanol, or other methylated compounds (47), were prominent in marker C fluids and very rare or absent in all other fluids (Table S2).

### Carbonic anhydrase.

At the high pH conditions of Lost City fluids, dissolved bicarbonate and carbonate are more stable than carbon dioxide, and the potential use of bicarbonate or carbonate as carbon sources has been explored in studies of continental sites of serpentinization (37, 48–52). Carbonic anhydrase catalyzes the reversible conversion between bicarbonate and carbon dioxide, which may enable cells to utilize bicarbonate obtained from the environment. *Methanosarcinaceae* MAG-1276 encodes a carbonic anhydrase that shares 59% to 85% amino acid identities with sequences found in three other MAGs from Lost City (classified as NPL-UPA2 and Bipolaricaulota) and in one MAG from the Hakuba Happo hot spring (53). These novel carbonic anhydrase sequences share only 35% to 41% amino acid identities with previously characterized proteins, e.g., the beta class carbonic anhydrases from Clostridium aceticum (Fig. S10). The Lost City carbonic anhydrase sequences retain each of the conserved residues highlighted by Smith and Ferry (54) for beta class carbonic anhydrases. In addition, the *Methanosarcinaceae* MAG includes a predicted high-affinity bicarbonate transporter (SbtA).

### Glycine reductase.

Glycine may be generated abiotically in high-H_2_ conditions or released as a primary thermogenic degradation production of biomass (55–58). The reduction of glycine to acetyl-phosphate is catalyzed by glycine reductase, which has been identified in metagenomes from multiple serpentinite-hosted springs (53). Seven of the Lost City MAGs encode glycine reductase, and in most of these genomes, glycine reductase (GrdEBCA) is in a gene cluster that includes selenium transferase (SelA), selenocysteine-specific elongation factor (SelB), and thioredoxin (TrxA) (Table S5), consistent with the gene organization of bacteria that conserve energy by reduction of glycine (59). Each of these MAGs also encodes a partial Wood-Ljungdahl pathway, including the key steps (formate-THF ligase and methenyl-THF cyclohydrolase dehydrogenase) that could potentially feed into the glycine reductase complex and thereby enable carbon fixation via the reductive glycine pathway (60). These initial observations remain to be verified with more complete genomic and experimental analyses but are consistent with early results from other serpentinization-associated environments (53).

### ATP synthase.

The production of ATP is catalyzed by the enzyme ATP synthase, which diverged into distinct archaeal and bacterial versions early in the evolution of life (61). A few of the bacterial MAGs in this study encode the archaeal form of ATP synthase (A-type or V-type) instead of the bacterial form (F-type). These include *Dehalococcoidia* MAG-844, Paceibacteria MAG-855, WOR-3 MAG-1066, and all three NPL-UPA2 MAGs (Table S5). Chloroflexi, Paceibacteria (previously named candidate phylum OD1), and NPL-UPA2 bacteria have also been observed to encode A-type ATP synthase in The Cedars, a continental serpentinite spring (33, 49). In addition, ATP synthase was completely absent in three of the Paceibacteria MAGs, as was the case for multiple Paceibacteria MAGs from The Cedars (49). *Natronincolaceae* MAG-1138 also lacks any ATP synthase genes, and its genomic content suggests an obligate fermentative lifestyle (supplemental material). Other genera within family *Natronincolaceae* include *Alkaliphilus* and *Serpentinicella*, which have been isolated from the Prony Bay hydrothermal field, which is also associated with serpentinization (62, 63).

## DISCUSSION

### Distinct zones of microbial activity in Lost City’s basement.

The massive edifice of Poseidon towers 60 m above the center of the Lost City hydrothermal field (Fig. 1). Alkaline hydrothermal fluids flow from the serpentinite basement and throughout the Poseidon structure, exiting at multiple locations across the field. The differing flow paths that lead to each location have distinct residence times (64) and produce distinct chemical and microbiological compositions of the venting fluids (9, 10, 18, 40).

For example, the venting locations marker 3 and Camel Humps sit only a few meters from each other at the summit of Poseidon, but the fluids venting from each structure appear to have taken different paths, which is reflected in their distinct microbial communities. Marker 3 fluids are dominated by a few archaeal and bacterial species that have the genomic potential to metabolize H_2_, formate, and sulfate. Genes encoding methanogenesis, sulfate reduction, and carbon fixation pathways are much more abundant in marker 3 fluids than genes encoding aerobic respiration pathways (Fig. 7; Fig. S12 to 15). In contrast, Camel Humps fluids host a diverse assemblage of bacteria capable of using oxygen, nitrate, and nitrite as oxidants. These taxonomic and metabolic patterns are generally similar between ribosomal gene and rRNA data sets (Fig. 2) and between metagenomes and metatranscriptomes (Fig. 3 and 7) from the same locations, indicating that the most abundant organisms in these fluids were likely to have been metabolically active at the time of sampling.

### Sulfate reduction is limited to a few taxa in the subseafloor.

Previous studies of Lost City hydrothermal fluids have revealed a consistent trend across the field in which the consumption of H_2_ and sulfate is correlated with the production of hydrogen sulfide (9–11). Therefore, SRB are expected to be widespread and metabolically active in the subsurface environments below the Lost City chimneys.

The metagenomic results presented here indicate a single, novel species of *Thermodesulfovibrionales* as the SRB that is most likely to be responsible for these trends. It dominates the fluids at marker C, marker 3, and Calypso, and accounts for most of the genes associated with sulfate reduction and H_2_ oxidation in these fluids. It also includes multiple formate dehydrogenases and various genes indicative of organic carbon oxidation (Table S5), suggesting metabolic flexibility that is not dependent on the availability of H_2_ and inorganic carbon.

The Lost City *Thermodesulfovibrionales* belong to a novel clade associated with deep subsurface environments and hot springs that shares only 82% to 87% nucleotide identities with the 16S rRNA genes of characterized *Thermodesulfovibrio* species (Fig. S7). Members of this clade of *Thermodesulfovibrionales* are also abundant and potentially important sulfate-reducing bacteria in chimneys of the “Old City” hydrothermal field along the Southwest Indian Ridge (22) and in highly alkaline borehole fluids associated with serpentinization of the Samail Ophiolite in Oman (5, 65, 66). In addition, sequences classified as *Thermodesulfovibrionales* (or at least the parent phylum *Nitrospirota*) have been detected in chimneys of the Prony Bay hydrothermal field (21), alkaline borehole fluids from the Coast Range Ophiolite (67), natural springs associated with the Zambales Ophiolite in the Philippines (68), and in the Hakuba Happo hot springs (34), but these short 16S rRNA sequences were generally found at low relative abundances and have not been compared with other *Thermodesulfovibrionales* in any phylogenetic analyses. Sulfate concentrations are much higher in borehole fluids from the Samail Ophiolite (up to 3.9 mM) and the Coast Range Ophiolite (up to 0.4 mM) compared with most natural springs associated with serpentinization (e.g., <0.02 mM in the Tablelands, Voltri Massif, and The Cedars) (32, 65, 67, 69). An exception is Ney Springs, where sulfate can be as high as 12.9 mM, but the potential SRB detected there did not include *Thermodesulfovibrionales* (70). In summary, this clade of *Thermodesulfovibrionales* is most abundant in marine sites of serpentinization (e.g., chimneys of Lost City and Old City) and in continental sites of serpentinization with elevated concentrations of sulfate (e.g., boreholes in Samail and Coast Range ophiolites).

### H_2_-fueled metabolism is limited to a few taxa in the subseafloor.

Lost City fluids contain copious quantities of H_2_ (1 to 7 mM, with subsurface concentrations predicted to reach 14 mM) (6, 40), which is expected to be a tremendous boost to life in the subseafloor. Surprisingly, only two taxa (*Thermodesulfovibrionales* and *Methanosarcinaceae*) that are abundant in Lost City fluids encode hydrogenases known to be associated with hydrogenotrophy. Therefore, the ability of the subseafloor ecosystem to be powered by H_2_ may depend on one species of bacteria and one species of archaea.

Another type of hydrogenase, known as membrane-bound hydrogenase (Mbh), was also detected in *Methanosarcinaceae*, ANME-1, and Bipolaricaulota genomes (Fig. 5). In *Thermococcus* and *Pyrococcus*, Mbh is responsible for H_2_ production during anaerobic, heterotrophic growth, and some bacteria use Mbh in coordination with FDH to convert formate into H_2_ (71, 72). In heterotrophic Bipolaricaulota, Mbh has been proposed to couple the production of H_2_ with ATP synthesis in coordination with the MvhAGD-HdrABC complex (73). In methanogens, the role of Mbh is unclear, but each of the methanogens that encode Mbh can utilize either formate or H_2_ as their sole source of electrons. Mbh has also been identified in a formate-consuming methanogen from the Samail Ophiolite, where it is proposed to be involved in the oxidation or reduction of ferredoxin (37). In H_2_-saturated Lost City fluids, biological production of additional H_2_ seems highly unfavorable, and the sequence divergence between the Lost City sequences and these previously characterized Mbh prevents any firm conclusions on whether they are more likely to catalyze the consumption or production of H_2_. Future metagenomic studies of other environments where these clades of *Methanosarcinaceae*, ANME-1, and Bipolaricaulota occur (21, 22, 49) would help to test whether these genes are uniquely adapted to the conditions of serpentinizing environments.

### Formate metabolism may operate via unknown mechanisms in the subseafloor.

Formate forms abiotically in the high-pH, reducing conditions of serpentinizing fluids, and is the second-most abundant form of carbon in Lost City fluids after methane and the second-most available reductant after dissolved H_2_ (4). Much of the biomass in Lost City chimneys is derived from carbon that originated in Earth’s mantle (9), most likely in the form of formate (10). Formate is the preferred substrate for methanogens in at least one other site of serpentinization where carbon dioxide is limiting (37). However, none of the taxa highlighted by this study contain any known formate transporters, and surprisingly few encode formate dehydrogenase (FDH), the enzyme that catalyzes the oxidation of formate. A remarkable exception is *Thermodesulfovibrionales*, which encodes three distinct forms of FDH.

A divergent, FDH-like sequence with unknown function was shared by four of the key taxa in this study (*Thermodesulfovibrionales*, *Methanosarcinacae*, ANME-1, and Bipolaricaulota). These sequences form a distinct clade that includes sequences from continental serpentinite springs, suggesting this gene represents a shared, unknown metabolic strategy in serpentinizing fluids (Fig. 6). At present, this phylogeny is sparse and lacks representatives of *Thermodesulfovibrionales* and *Methanosarcincaceae* from other sites of serpentinization where those taxa are abundant (5, 21, 22, 74). For example, no *Methanosarcinaceae* MAGs from other serpentinizing environments have yet been reported. Therefore, ongoing and future metagenomic studies of these environments will soon reveal whether the divergent form of FDH reported here is widespread among taxa and environments associated with serpentinization.

In the highly reducing conditions of Lost City fluids, biosynthetic pathways are more energetically favorable than in typical environments, and the synthesis of some biomolecules can even be energy-yielding (58, 75). Therefore, the ability to incorporate formate directly into metabolic pathways, rather than first oxidizing it to carbon dioxide, could be a competitive advantage in Lost City’s basement, where formate is 100 to 1,000 times more abundant than carbon dioxide (4). Potential evidence for this hypothesis is the prevalence of partial and complete Wood-Ljungdahl pathways among Lost City bacteria (Table S5). Eight of these genomes do not encode a known FDH, suggesting they may be able to use formate, rather than carbon dioxide, as the substrate for carbon fixation and perhaps acetogenesis. Some acetogens can use formate as their sole source of energy and carbon, although FDH may be still required to supply carbon dioxide as an electron acceptor (76).

In the absence of FDH, pyruvate formate lyase (PflD), which is encoded by some of the same genomes with partial Wood-Ljungdahl pathways (Bipolaricaulota, NPL-UPA2, and *Dehalococcoidia*), might catalyze the reduction of formate directly into acetyl-CoA and pyruvate (77). However, this activity has only been demonstrated in Escherichia coli, and its relevance to these taxa in the unusual environmental conditions of Lost City requires further research.

### Conclusions.

This study has highlighted multiple examples of metabolic strategies shared among the archaea and bacteria most likely to inhabit subsurface habitats underlying the Lost City hydrothermal field. These shared strategies appear to be advantageous for life in environments that are rich in H_2_ (e.g., hydrogenases), provide a steady supply of simple organic molecules (e.g., formate dehydrogenase, pyruvate formate lyase, and glycine reductase), lack carbon dioxide (e.g., carbonic anhydrase), and make typical ATP synthesis too difficult or unnecessary (e.g., the absence of ATP synthase in some MAGs).

Many of the predicted proteins associated with these metabolic strategies are not closely related to any previously characterized enzymes, but are shared by diverse archaea and bacteria in Lost City and other sites of serpentinization (e.g., Old City, Prony Bay, The Cedars, Samail Ophiolite, Coast Range Ophiolite, Hakuba Happo, and Voltri Massif), strongly suggesting the influence of horizontal gene transfer among these systems. The functions of these proteins are mostly unknown and require further study, but the results presented here indicate they are likely to be important clues for understanding the ecology, physiology, and evolution of microbes adapted to these conditions.

If potential extraterrestrial habitats are evaluated for their ability to support a robust ecosystem over geological time scales (78), then it is critical to identify and understand the metabolic pathways of key organisms that form the foundations of ecosystems that are potentially relevant for astrobiology. All ecosystems on the surface of the Earth are based on autotrophs that rely on the availability of sunlight and carbon dioxide. The most promising extraterrestrial habitats in our solar system (79–82), however, are dark, rock-hosted environments where simple organic molecules may be more biologically available than carbon dioxide. The organisms and metabolic pathways highlighted by this study can help us to understand the biological advantages and limitations of such conditions.

## MATERIALS AND METHODS

### Collection of hydrothermal fluid samples.

Hydrothermal fluid samples were collected from actively venting chimneys at the Lost City hydrothermal field (Fig. 1) using ROV *Jason* during the 2018 Lost City expedition aboard R/V *Atlantis* (AT42-01). On the seafloor, venting fluids were slowly pumped through 0.2 μm Millipore Sterivex cartridge filters or into acid-washed Kynar bags with the HOG sampler (83). Samples intended for RNA extractions were collected into 2 L Kynar bags containing 67 mL of a stop solution (97.5% 200 proof ethanol, 2.5% TRIzol LS; Thermo Fisher). Fluid temperatures were monitored in real-time during sampling with a probe embedded into the sampler intake. Immediately upon shipboard recovery of ROV *Jason*, all Sterivex filters were stored at −80°C. Kynar bags were subsampled for shipboard aqueous chemistry measurements (pH, magnesium, sulfate, and hydrogen sulfide) according to standard methods (84) and filtered through Sterivex filters, which were subsequently frozen at −80°C. The estimated precision of the analyses is 3% for magnesium, 2% for sulfate, and 4% for hydrogen sulfide (all as relative standard deviation). All frozen filters were shipped overnight to the University of Utah on dry ice or in liquid nitrogen vapor shippers.

### Cell counts.

Aliquots of all fluid samples were preserved in 3.7% formaldehyde and stored at 4°C. In the Anderson lab (Carleton College), preserved fluids were filtered onto 0.22 μm black polycarbonate filters and stained with DAPI (85). Cells were enumerated directly with an epifluorescence microscope, and a minimum of 20 fields of view were counted per sample. The median number of cells per field of view was recorded as the result for that sample, and the median value among all replicates is reported as the value for each location in Table 1.

### Bags supplemented with formate or methane.

On each dive, some of the 2 L Kynar bags were supplemented with ^13^C-enriched bicarbonate, formate, or methane (Cambridge Isotope Laboratories) to test for conversion of these compounds into DIC or methane inside the Kynar bags. Thus, these Kynar bags served as seafloor incubation experiments. In addition, each bag contained 0.2 g of dithiothreitol, intended as a redox buffer. Upon shipboard recovery, the bags were subsampled and processed for DNA sequencing as described above, and additional aliquots of the fluid sample were collected for analysis of ^13^C enrichment of methane and DIC. In the Lang lab (University of South Carolina), the ratio of ^13^C/^12^C of headspace CO_2_ was analyzed using a Thermo Scientific GasBench-Delta V Plus isotope ratio mass spectrometer (IRMS). The ^13^C/^12^C of CH_4_ was analyzed with a Thermo Scientific Gas Chromatograph-IsolinkII-IRMS equipped with an Agilent GS - CarbonPlot column (30 m × 0.320 mm i.d., 1.50 um film thickness). Amplicon sequencing was conducted on each fluid sample separately, and no consistent differences in microbial community composition were detected among fluids collected in incubation bags compared with *in situ*-filtered Sterivex filters (Table S2). Nevertheless, separate metagenome libraries were constructed with DNA prepared from *in situ*-filtered Sterivex filters and from incubation bags (Table S1).

### Analysis of 16S rRNA ASVs.

Sequencing of amplicons generated from 16S rRNA genes and cDNA was performed at the Genomics Core Facility at Michigan State University on an Illumina MiSeq instrument using dual-indexed Illumina fusion primers targeting the V4 region of the 16S rRNA gene (86). ASVs were inferred from 16S rRNA amplicon sequences with DADA2 v. 1.10.1 (87) after removal of primer sequences with cutadapt v. 1.15 (88). Sequences from different sequencing runs were processed with DADA2 separately, and then the resulting ASVs from all sequencing runs were merged to form a final, nonredundant count table of ASVs. Potential contaminants were removed with the decontam package (89) using both the “frequency” and “prevalence” modes. In the “frequency” mode, ASVs were removed if their counts were significantly correlated (*P* < 0.02) with DNA concentration (as measured by Qubit fluorometric quantification, Thermo Fisher). In the “prevalence” mode, ASVs were removed if they were significantly more likely (*P* < 0.02) to be present in one of three likely contamination sources (ambient lab air, surface seawater, or DNA extraction blanks) than in any sample of hydrothermal fluid. Samples of ambient air in the R/V *Atlantis* shipboard laboratory and our laboratory at the University of Utah were obtained as previously described (14). Samples of surface seawater were obtained during a previous study at the same location above the Atlantis Massif (14) and also from the shipboard laboratory “tap” water produced from surface seawater by the R/V *Atlantis*. DNA extraction blanks were obtained by Motamedi et al. by subjecting sterile Sterivex filters to the DNA extraction protocol described above (14). All contamination control samples were sequenced on the same sequencing runs as described above for the hydrothermal fluid samples. A total of 1,823 ASVs (9% of all ASVs) representing 109,824 sequence counts (2% of all sequence counts) were removed from downstream analyses by decontam. These removed ASVs are provided in Table S2.

Taxonomic classification of all ASVs was performed with DADA2 using the SILVA reference alignment (SSURefv132) and taxonomy outline (90, 91). Bubble plots of ASVs were drawn with ggplot2 using proportional abundances. The ordination plot was generated with phyloseq v1.26.1 (92) using an unconstrained NMDS ordination of Morisita-Horn dissimilarity values. The stress of the fit to two dimensions was 0.05 after 20 tries without a convergent solution. Very similar results were produced with MDS and CCA ordinations and with Bray-Curtis dissimilarity values.

Differential abundances of ASVs between the marker 3 and Camel Humps locations were calculated with DESeq2 (93) as implemented by phyloseq (92). ASVs with variance <10^−5^ were filtered out prior to the test, and those ASVs with an adjusted *P*-value <0.05 were considered to be differentially abundant.

### Sequencing of metagenome libraries.

Metagenome libraries were constructed with size-selected, sonicated DNA fragments of 500 to 700 bp with the NEBnext Ultra DNA II library kit for Illumina (E7645S), as previously described (94). Paired-end sequencing (2 × 125 bp) of metagenomic libraries was conducted at the University of Utah High-Throughput Genomics Core Facility at the Huntsman Cancer Institute with an Illumina HiSeq 2500 platform. Sequencing libraries (25 pM) were chemically denatured and applied to an Illumina HiSeq v4 paired end flow cell using an Illumina cBot. Hybridized molecules were clonally amplified and annealed to sequencing primers with reagents from an Illumina HiSeq PE Cluster Kit v4-cBot. Following transfer of the flowcell to an Illumina HiSeq 2500 instrument (HCS v2.2.38 and RTA v1.18.61), a 125 cycle paired-end sequence run was performed using HiSeq SBS Kit v4 sequencing reagents (FC-401-4003).

### Sequencing of metatranscriptome libraries.

Only two samples (one from marker 2 and one from Sombrero) contained sufficient total RNA to attempt metatranscriptome sequencing. The two metatranscriptome libraries were constructed and sequenced by the University of Utah High-Throughput Genomics Core Facility at the Huntsman Cancer Institute. Total RNA was hybridized with NEBNext rRNA Depletion Solution Bacteria (E7850L) to substantially diminish rRNA from the samples. Stranded RNA sequencing libraries were prepared using the NEBNext Ultra II RNA Library Prep Kit for Illumina (E7770L). Purified libraries were qualified on an Agilent Technologies 2200 TapeStation using a D1000 ScreenTape assay. The molarity of adapter-modified molecules was defined by quantitative PCR using the Kapa Biosystems Kapa Library Quant Kit. Individual libraries were normalized to 10 nM, and equal volumes were pooled. Sequencing libraries were chemically denatured and applied to an Illumina NovaSeq flow cell using the NovaSeq XP workflow (20043131). Following the transfer of the flowcell to an Illumina NovaSeq 6000 instrument, a 150 cycle paired-end sequence run was performed using a NovaSeq 6000 S4 reagent kit v1.5 (20028312).

### Quality control and taxonomic classification of metagenome and metatranscriptome sequences.

Demultiplexing and conversion of the raw sequencing base-call data were performed through the CASAVA v1.8 pipeline. Adapter sequences and PhiX were removed from all reads with BBDuk (part of the BBTools suite, v35.85 [95]). Quality trimming was performed with our seq-qc package (https://github.com/Brazelton-Lab/seq-qc) as previously described (94). Each library yielded 22 to 192 million reads (for a total of 1.4 billion reads among all metagenome libraries) after these quality control steps, representing 58% to 82% of the number of raw reads. The two metatranscriptome libraries yielded 249 million reads (marker 2) and 467 million reads (Sombrero) after quality filtering, representing 82% and 88%, respectively, of the original raw reads. rRNA sequences were identified in the two metatranscriptomes with SortMeRNA (96), resulting in the removal of 77% of reads from the marker 2 metatranscriptome and 90% of reads from the Sombrero metatranscriptome. Quality-filtered, unassembled reads were assigned taxonomy with Kaiju and the NCBI nr+euk database (97). Kaiju was unable to classify 46% to 86% of reads, with the lowest percentage of unclassified reads in metatranscriptomes and the highest percentage of unclassified reads in the two marker 3 metagenomes and one Sombrero metagenome (Table S3). An interactive Krona plot is provided in the Zenodo-archived GitHub repository (doi:10.5281/zenodo.5798015).

### Metagenomic assembly.

Sequences from metagenomic libraries were assembled with Megahit v1.1.1 (98), using kmers of 27 to 127. A pooled “all fluids” Megahit assembly was performed with metagenomic reads from all 13 libraries (representing 37 fluid samples collected in Sterivex filters and Kynar bags; see Table S1). Genes were predicted with Prodigal v2.6.3 (99) in meta mode. Predicted protein sequences were queried against the KEGG release 83.2 prokaryotes database with Diamond v0.9.14 (100).

In addition to the “all fluids” Megahit assembly, chimney-specific assemblies were conducted with metaSPAdes v3.13.0 (101) as implemented by the KBase platform (kb_SPAdes v.1.2.4) (102). A chimney-specific assembly with reads obtained from marker 3 was performed with Megahit v1.1.1 because metaSPAdes repeatedly failed when it attempted to assemble marker 3 reads. An overview of the metagenomic analysis workflow is illustrated in Fig. S3.

### Binning of MAGs.

Binning of MAGs from the “all fluids” and marker 3 Megahit assemblies was conducted with BinSanity using the Binsanity-lc workflow v0.2.6.2 and a minimum contig size of 3 kb (103). Binning of MAGs from the chimney-specific metaSPAdes assemblies was conducted with MaxBin2 v2.2.4 (kb_maxbin v.1.1.1) (104), MetaBAT2 v2.2 (kb_metabat v.2.3.0) (105), and DAS Tool v1.1.2 (kb_das_tool v.1.0.6) (106). The ANME-1 MAG was reconstructed from a Megahit assembly of Calypso reads with MetaBAT2 and DAS Tool. MAGs were assigned taxonomic classifications with GTDB-Tk v1.5.1 (reference data version r202; [107]). Individual contigs were assigned taxonomic classifications with MMseqs2 (108). Gene products were predicted by annotation with Prokka v1.14.5 and its default databases (109) with further function prediction provided by GhostKOALA v2.2 (110). Protein identifications and predicted functions were supplemented by results from InterProscan 5 (v5.52-86.0) (111), HydDB (112), FeGenie (113), and dbCAN2 (114). Completeness and contamination of initial automated MAGs were estimated with CheckM v1.0.5 (115), and the completeness and redundancy of the final refined MAGs were estimated with anvi’o v7 (116). Selected MAGs were refined by reassembly with metaSPAdes v3.11.1 using reads mapped to MAGs and contigs with matching taxonomic classifications, followed by manual curation in anvi'o v7 (117) according to the differential coverage of contigs among samples.

### Coverages of genes, contigs, and MAGs.

Quality-filtered metagenome and metatranscriptome sequences were mapped to the “all fluids” Megahit assembly with Bowtie2 v2.3.2 (118). Bowtie2 mapping rates to the “all fluids” assembly were 67% to 71% for Camel Humps, 82% to 89% for Sombrero1, 60% to 71% for Sombrero2, 95% for marker 3, 67% to 74% for Calypso, and 56% to 60% for marker 2. Bowtie2 mapping rates to the “all fluids” assembly were 21% before rRNA removal and 14% after rRNA removal for the marker 2 metatranscriptome and 83% before rRNA removal and 74% after rRNA removal for the Sombrero1 metatranscriptome. Artificial duplicates were removed from the bam files using the MarkDuplicates function of Picard Tools v2.17.8 (119).

The coverage for each predicted protein was calculated as TPM with count_features v1.3.0, part of our seq-annot package (94). TPM is a proportional unit (multiplied by one million for convenience) that is normalized to the length of each predicted protein sequence as well as to the total library size. Coverages of both metagenomes and metatranscriptomes were calculated in the same way and reported in the same units (TPM), although the metagenomic coverages reflect fragments instead of transcripts (94).

The coverage of each MAG was calculated as the weighted sum of the normalized, proportional coverages (in TPM) of its member contigs. The contig coverages were obtained by mapping all unassembled reads against the reassembled, refined MAGs with Bowtie2 and then calculating the average coverage per position of each contig with the genomecov command (with the option -pc) in bedtools v2.30.0 (120). Normalized coverages in units of TPM were calculated by dividing the average coverage per position by the total number of read pairs for that library.

### Phylogenetic analyses.

Phylogenetic trees of 16S rRNA genes were constructed with alignments obtained from the SILVA alignment server (121) and RAxML v8.2.11 (122) using the gamma model of rate heterogeneity and the “-f a” option to construct 100 rapid bootstrap searches and 20 maximum likelihood searches. The *Thermodesulfovibrionales* ASV from the Samail Ophiolite, Oman (65) was generated with DADA2 as described above with reads accessed via SRA accession SRR5000240. Phylogenies of McrA, GrdB, and carbonic anhydrase were constructed with Clustal Omega alignments (123) and RAxML as described above but with automated selection of the rate model. All sequences and accessions are provided in the Zenodo-archived GitHub repository accessible via doi:10.5281/zenodo.5798015.

### Data availability.

Amplicon sequences are available via NCBI BioProject PRJNA672129, and metagenome and metatranscriptome sequences are available via BioProject PRJNA779602. MAGs are associated with the same BioProject and are individually accessible via BioSamples SAMN23474158 - SAMN23474187. In addition, GenBank accessions are listed for each MAG in Table S4. Protocols, metadata, and additional data are provided in a Zenodo-archived GitHub repository accessible via doi:10.5281/zenodo.5798015, and on the BCO-DMO page for project 658604: https://www.bco-dmo.org/project/658604.
