# Supplementary material for: Metabolic Strategies Shared by Basement Residents of the Lost City Hydrothermal Field
Source: Appl Environ Microbiol. 2022 Aug 11;88(17):e00929-22. doi: 10.1128/aem.00929-22 (PMC9469722; doi:10.1128/aem.00929-22)
Supplement: Supplemental file 1 — Supplemental text and Fig. S1 to S15. Download aem.00929-22-s0001.pdf, PDF file, 6.2 MB [file aem.00929-22-s0001.pdf]

Supplemental Text: Additional Results

Brazelton et al. "Metabolic strategies shared by basement residents of the Lost City hydrothermal field"

DETAILED COMPARISONS OF HYDROTHERMAL FLUID SAMPLES

Overview of sampling locations

The present study included hydrothermal fluid samples from seven chimney locations: Camel Humps, Sombrero, Marker 3, Marker C, Calypso, Marker 2, and Marker 8 (**Table 1; Supplemental Table S1**). Several fluid samples collected from the Beehive chimney, where the highest fluid temperatures have been measured, yielded only low-quality DNA sequences and are not included in this study. The Sombrero site was sampled on two different ROV *Jason* dives, and samples from the separate dives are labeled as Sombrero1 or Sombrero2 when appropriate. Fluid samples collected from Markers C, 2, 3, and 8 were included in an early microbial diversity study (1), but microbial diversity data from the other chimneys are reported here for the first time.

Markers 3 and C

The strong similarity of community compositions from Markers 3 and C is remarkable considering that fluids from Marker 3 were sampled at much lower temperatures (<20 ºC) than those from Marker C (up to 80 ºC). Temperatures exceeding 55 ºC were measured at Marker 3 during the same dive, but the temperatures of the fluid samples (i.e. measured in-line, during sampling) used for DNA-based analyses were lower, most likely due to cooling of the fluids as they exit the chimney. In addition, the pH, sulfate, sulfide, and magnesium levels of Marker 3 fluids are more similar to those of seawater, compared to Marker C fluids. These results suggest that the cooler fluids venting from Marker 3 experience more dilution with ambient seawater

compared with the warmer fluids venting from Marker C, but that they share a common subsurface source, which may explain the similar microbial compositions.

Marker 3 fluids were rich in metagenomic sequences classified as family *Methanosarcinaceae,* which includes the dominant archaeal phylotype previously detected in Lost City chimneys (2–4). Archaeal sequences were much more abundant in metagenomes than in the 16S rRNA amplicon libraries. For example, *Methanosarcinaceae* is the most abundant family in Marker 3 metagenomic reads (1-2% of all reads and 20-21% of all reads that could be classified to the family level by Kaiju; **Supplemental Table S3**), even though *Methanosarcinaceae* ASVs have lower relative abundance than several bacterial ASVs in the same samples, suggesting a bias against archaeal sequences in the ASV dataset.

Venting fluids collected near Markers C and 3 were particularly enriched in taxa representing potential sulfate-reducing bacteria (SRB). *Thermodesulfovibrionia* and *Desulfotomaculum* ASVs dominated these fluids, but they were much less abundant in the RNA fraction of the Marker C sample (**Figure 3**). ASVs classified as *Desulfocapsa* were abundant in Marker 3 fluids, present in the RNA fraction from Marker C, and absent in the DNA fraction from Marker C. *Desulfobulbus* ASVs were absent in both Markers C and 3 except for the RNA fraction from Marker C, and they were prevalent in Sombrero fluid samples. *Desulfobulbus* and *Desulfocapsa* both belong to the family *Desulfobulbaceae*, which had 30-fold less coverage in Marker 3 metagenomes than *Methanosarcinaceae* and 8-fold less coverage than the *Nitrospiraceae* family that includes *Thermodesulfovibrio* (**Supplemental Table S3**).

Marker 3 metagenomes were also distinctive in their high proportions of Candidatus Patescibacteria (**Supplemental Figure S2**). Patescibacteria were rare in all samples except for Marker 3 fluids, where they represented 33-36% of all classified sequences (**Supplemental**

Table S3). Hundreds of ASVs were classified as Patescibacteria (predominantly Paceibacteria and Gracilibacteria), but they did not represent a large fraction of the counts in any of the fluid samples (**Supplemental Table S2**).

Camel Humps and Sombrero

Fluids venting from Camel Humps contained a remarkably even distribution of ASVs that included *Sulfurovum*, *Sulfurospirillum, and Thiomicrorhabdus* at similar abundances as taxa typically associated with ambient seawater (e.g. *Alteromonas*, *Roseobacter, Halomonas*). This community composition strongly contrasts with that of Marker 3, even though both locations are at the summit of the central Poseidon structure (**Figure 1**). Of the 100 ASVs most common in Camel Humps fluids, 70 of these were significantly less abundant in Marker 3 fluids (**Supplemental Table S2**). The differences between Camel Humps and Marker 3 cannot be explained by mixing of the same hydrothermal fluid with ambient seawater because the bacteria at Camel Humps belong to lineages associated with hydrothermal environments (e.g., *Sulfurovum*, *Sulfurosprillum*, *Thiomicrorhabdus*), not ambient seawater. Moreover, the temperatures measured during the sampling of Camel Humps fluids were higher than those of Marker 3 fluids, which is the opposite of what would be expected if dilution by seawater had been responsible for the observed trends. Instead, these results suggest a difference in the subsurface source of the hydrothermal fluids venting from these locations. Although Marker 3 and Camel Humps are located next to each other, they are visually distinct chimney structures that could potentially host fluids venting from distinct subsurface sources (**Supplemental Figure S2**). Alternatively, the distinctive assemblage of taxa at Camel Humps may represent biofilm communities that were flushed into nearby venting fluids, swamping the less dense organisms derived from the subsurface. The two explanations are not mutually exclusive, and both indicate that the fluids collected from Camel Humps during this study are not representative of the subseafloor.

The Sombrero chimney is located on a ridge stretching from the main vent field toward the eastern wall of the Atlantis Massif (**Figure 1**), and it was sampled on two separate ROV *Jason* dives, with the second dive (Sombrero2) collecting fluids with higher temperatures and lower sulfate concentrations than the first dive (Sombrero1) (**Table 1**). Despite these different measurements at the time of sampling, the overall microbial composition was remarkably consistent across all Sombrero samples ranging in temperature from 10 – 74 ºC (**Figure 1**). Minor differences are nevertheless visible (**Figure 2**); for example, *Thiomicrorhabdus* and *Sulfurospirillum* dominated Sombrero fluids with lower temperature, higher sulfate, and lower sulfide. Warmer and more sulfidic Sombrero fluids included greater proportions of taxa that were also abundant in fluids from Markers 3 and C (**Figure 2**).

Markers 2 and 8

The most sulfidic fluids were collected from chimneys near Marker 2 and Marker 8, which are located on the western edge of the Lost City hydrothermal field (**Table 1**; **Figure 1; Supplemental Figure S1**), and their microbial compositions were distinct from all other fluids. One of the fluid samples from Marker 2 was dominated by a single ASV identical to the 16S rRNA gene of *Alteromonas macleodi* (**Supplemental Table S2**), a ubiquitous marine bacterium (5), suggesting substantial dilution of the sample with ambient seawater. Other samples from Marker 2 also contained bacteria that have been previously associated with sulfur oxidation in Lost City chimney biofilms, including the genera *Sulfurovum*, *Sulfurospirillum*, and *Thiomicrorhabdus* (3, 6, 7). Therefore, they are likely to be adapted to chimney habitats where sulfidic hydrothermal fluids are mixing with oxic seawater, and they are probably not representative of microbial communities inhabiting anoxic, subsurface environments. The ribosomal RNA fraction of Marker 2 fluids contained elevated relative abundances of a *Sulfurospirillum* ASV and a *Thiomicrorhabdus* ASV (**Figure 2**). Microbial taxa detected in fluids

collected near Marker 8 were broadly similar to those of Marker 2 fluids, except that they were dominated by one *Sulfurovum* ASV that was rare in Marker 2 fluids (**Figure 2**).

Calypso

The Calypso chimney sits on the eastern wall of the Atlantis Massif, approximately 75 m from the large Poseidon edifice that dominates the Lost City field (**Figure 1**). The fluids venting from Calypso had higher sulfide concentrations than the fluids from Markers 3 and C, but less sulfide than in Markers 2 and 8 (**Table 1**). The overall microbial community structure resembled that of Sombrero fluids (**Figure 1**), although the ASVs with the highest counts in Calypso were also common in Markers 3 and C (**Figure 2**). The top *Thermodesulfovibrionia* ASV in Calypso fluids differed by one base from the ASV that dominated the fluids from Markers C and 3. This ASV was further enriched in the RNA fraction from Calypso, suggesting that it could have been metabolically active prior to sampling.

ASVs classified as the ANME-1b group of archaeal methanotrophs were most abundant in fluids from Calypso (**Figure 2**). ANME-1 sequences were rare or absent in almost all other fluid samples except for a few samples from Markers C and 3 and the RNA (but not DNA) fraction from one sample of fluids at Marker 2. These results are consistent with our previous studies that documented very high relative abundances of ANME-1 in cooler chimneys at the periphery of the field and trace levels of ANME-1 DNA in hot chimneys of the central Poseidon complex (1, 3).

Calypso fluids were rich in Chloroflexi ASVs and metagenomic sequences primarily classified as class *Dehalococcoidia* (**Supplemental Tables S2-S3)**, but ASVs classified as *Anaerolineae*, TK10, and KD4-96 were also present at low levels. The Chloroflexi MAG from Lost City biofilms

previously reported in (7) is a member of the *Anaerolineae*. *Dehalococcoidia* ASVs were generally most abundant in Calypso fluids, but most ASVs were broadly distributed (**Figure 2**).

DETAILED DESCRIPTIONS OF METAGENOME-ASSEMBLED GENOMES (MAGs)

Methanosarcinaceae MAG

The predominant *Methanosarcinaceae* ASV was classified as genus *Methanosalsum* in the SILVA database, but our phylogenies of 16S rRNA and mcrA indicate that the Lost City *Methanosarcinaceae* are not monophyletic with any previously characterized genera (**Supplemental Figure S5**). Over 254 bases, the *Methanosarcinaceae* ASV is 94% similar to the 16S rRNA gene of *Methanococcoides methylutens* and 95.7% similar to that of *Methanohalophilus mahii*.

The *Methanosarcinaceae* MAG contained 1276 coding sequences (CDS) and was estimated to be 84% complete with 1% redundancy. It shares 99% average nucleotide identity (ANI) with the MAG we previously recovered from a Lost City biofilm (7). It is most abundant in Marker 3 fluids (**Figure 3**), and it is more abundant in those Sombrero fluids that show more mixing with seawater (lower temperature, more sulfate, less sulfide), consistent with the distribution of *Methanosarcinaceae* ASVs (**Figure 2**).

Methanosarcinaceae MAG-1276 has a remarkably low GC content (29%), which may help explain why the first metagenomic studies of Lost City biofilms recovered surprisingly few archaeal sequences (6, 8). It encodes the core pathway for methanogenesis from carbon dioxide (**Supplemental Table S5**). It contains predicted sequences for F_{420}-reducing hydrogenase (FrhAB), which is required by all methanogens that reduce carbon dioxide with H_2

(9). In addition, two genes predicted to encode subunits of Ech hydrogenase (EchCE) are present. FrhB and EchCE are located on the same contig as a multicomponent Na^+:H^+ antiporter (MrpACBD) and the MAG's only gene annotated as a subunit of NADH-quinone oxidoreductase (NuoH).

The MAG encodes AMP-forming acetyl-CoA synthetase, as previously reported for *Methanosarcinaceae* in Lost City biofilms (7), which may enable acetoclastic methanogenesis. It also encodes MtaA and MtaC (**Supplemental Table S5**), two of the three proteins that enable the use of methanol as a substrate for methanogenesis in some methanogens.

Methanosarcinaceae MAG-1276 contains a complete 14-gene cluster (mbhA-N) encoding membrane-bound hydrogenase (**Figure 5; Supplemental Figure S8**). The same gene cluster, with conserved synteny, is also found in methanogens belonging to the order *Methanomicrobiales* (e.g. *Methanospirillum hungatei*) and in heterotrophs of the order *Thermococcales* (e.g. *Thermococcus kodakarensis*) (10). The mbhL subunits from these methanogens have only 42-45% identities with the Lost City mbhL sequences reported here, which have greater similarly (~49% identities) to mbhL sequences from *Thermococcus*.

As we reported previously for chimney biofilms (11), the *Methanosarcinaceae* MAG encodes a formate dehydrogenase (FDH) that is similar to that of *Methanolobus* species, which are unable to use formate as a carbon source. The contig encoding this FDH also encodes one subunit of F_{420}-non-reducing hydrogenase (MvhD) and heterodisulfide reductase (HdrABC).

No known transporters for formate were identified in this MAG, but one gene was predicted to encode a member of the oxalate:formate antiporter family. In *Oxalobacter formigenes*, this transporter enables uptake of oxalate with export of formate (12). Its presence was also

observed in a formate-utilizing methanogen, which lacks any other formate transporters, in hyperalkaline groundwaters of the Samail Ophiolite (13). Potentially homologous oxalate:formate antiporters were also identified in two NPL-UPA2 MAGs and two *Natronincolaceae* MAGs from Lost City fluids. The four bacterial sequences shared 59-63% amino acid identities with the *Methanosarcinaceae* sequence.

Methanocellales MAG

A MAG classified as order *Methanocellales* (838 CDS; 84% complete with 1% redundancy) was only present in Calypso fluids (**Figure 3**). Curiously, no ASVs were classified as *Methanocellales*, and no previous studies have identified any methanogen taxa in Lost City samples other than *Methanosarcinaceae*.

Methanocellales MAG-838 encodes the key enzyme CODH/ACS, but it has an incomplete pathway for methanogenesis, including only four of the first five steps (FwdABCDFG, Ftr, Mtd, and Mer) and lacking all other steps, including the proteins required for methane production (methyl-coenzyme M reductase and heterodisulfide reductase) (**Supplemental Table S5**). Evidence for acetate utilization includes a predicted sequence for AMP-forming acetyl-CoA synthetase, which shares 73% amino acid identities with the homolog in the *Methanosarcinaceae* MAG, and a cation/acetate symporter (ActP). The MAG includes one [NiFe] hydrogenase and the Rnf complex (RnfCDGEAB), an energy-conserving ferredoxin:NAD^+-oxidoreductase (14).

ANME-1 MAG

A MAG classified as ANME-1 (1099 CDS; 88% complete with 4% redundancy) was most abundant in Calypso fluids as well as the Marker 2 metatranscriptome, even though ANME-1 was very rare in the Marker 2 metagenomes. As expected for ANME-1 archaea, the Lost City

ANME-1 MAG contains the core methanogenic pathway (**Supplemental Table S5**). The absence of cytochromes and presence of hydrogenases in this MAG was noted by (15) as consistent with the genomic features of the so-called "freshwater" clade of ANME-1, for which the genus "Candidatus Methanoalium" was proposed. One of the shared features within this clade, including the Lost City ANME-1 MAG, is a novel HdrABC-MvhADG complex (15), which is involved in the transfer of electrons derived from H_2 in methanogens.

The Lost City ANME-1 MAG contains the core methanogenic pathway (**Supplemental Table S5**), including F_{420}-dependent methylenetetrahydromethanopterin reductase (Mer). This gene is required for methanogenesis from carbon dioxide, but it is typically absent in ANME genomes, with at least one exception previously reported (16). The MAG lacks all but one of the subunits of N^5-methyl-H_4MPT:coenzyme M methyltransferase (Mtr), which catalyzes the penultimate step of methanogenesis (and putatively the second step of reverse methanogenesis). It is present in most but not all ANME-1 genomes (15).

The ANME-1 MAG-1 also encodes the complete mbhA-N gene cluster for membrane-bound hydrogenase (**Figure 5; Supplemental Figure S8**), and each predicted gene in the cluster has the greatest similarity to the homolog in the *Methanosarcinaceae* MAG than to any other sequences in public databases. It lacks any established genes for FDH, but it includes a divergent FDH-like sequence that appears to be homologous to those found in other Lost City MAGs (**Figure 6; Supplemental Figure S9**). Distant homologs of FDH have been previously identified in ANME genomes, but they do not share significant sequence similarity with the FDH-like sequences reported here.

Bipolaricaulota MAGs

ASVs classified as Bipolaricaulota (named Acetothermia in the SILVA database) clustered into three distinct clades (**Supplemental Figure S6**) that correspond to the taxonomic classifications of three distinct Bipolaricaulota MAGs. The most abundant of the three Bipolaricaulota MAGs (1207 CDS; 86% complete with 0% redundancy) was classified by GTDB as species UBA7950 within class Bipolaricaulia, and it shares 99% ANI with the GTDB reference genome, which was assembled by (17) with sequences from a previously published Lost City biofilm metagenome (11). Bipolaricaulota MAG-1207 is most abundant in Sombrero and Calypso, especially the Sombrero metatranscriptome (**Figure 3**). It encodes a nearly complete glycolysis pathway, an incomplete TCA cycle, an incomplete Wood-Ljungdahl pathway, the Rnf complex, and pyruvate formate-lyase (PflD) (**Supplemental Table S5**). The key enzyme of the Wood-Ljungdahl pathway, CODH/ACS, is represented by the subunits AcsABC, although at least one subunit (AcsB) appears to be replaced by the archaeal form (CdhC), according to GhostKoala results. A thorough phylogenetic analysis of CODH/ACS subunits in Lost City MAGs is outside the scope of the present study, but this initial observation is consistent with observations in MAGs from other serpentinization-associated environments (18).

A second Bipolaricaulota MAG (1260 CDS; 89% complete with 1% redundancy) was classified as family UBA9294 within order UBA7950, and it shares only 77% ANI with the closest reference in GTDB (**Supplemental Table S4**). Bipolaricaulota MAG-1260 has a similar complement of genes as in MAG-1207, including an incomplete Wood-Ljungdahl pathway (CODH/ACS subunits AcsBC and CooC) and the Rnf complex, though it lacks pyruvate formate-lyase and glycine reductase.

Neither of these Bipolaricaulota MAGs contains any known hydrogenases or formate dehydrogenases. A divergent FDH-like sequence that appears to be homologous with those in the *Methanosarcinaceae*, ANME-1, and *Thermodesulfovibrionales* MAGs was observed in

multiple initial BinSanity bins classified as Bipolaricaulota (**Figure 6**, but it was not included in the three re-assembled, refined Bipolaricaulota MAGs. The most similar sequence in the NCBI NR database was from a MAG assembled by (17) from a Voltri Massif serpentinite spring (19).

A third Bipolaricaulota MAG (1503 CDS; 92% complete with 1% redundancy) was classified as genus UBA3574 within family Bipolaricaulaceae. It encodes a nearly complete Wood-Ljungdahl pathway (including CODH/ACS subunits AcsBCDE and CooC) and the NADP-dependent formate dehydrogenase (FdhA) that is typical of acetogens. It does not encode the Rnf complex, but it contains genes encoding all subunits of the heterodisulfide reductase complex MvhAGD-HdrABC. It encodes a [NiFe] hydrogenase (HoxYH) and at least a partial gene cluster for membrane-bound hydrogenase, including the large catalytic subunit MbhL (**Figure 5; Supplemental Figure S8**). The predicted MbhL sequence is most closely related to two Bipolaricaulota MAGs from hydrothermal systems: the Mid-Cayman Rise (20) and Guaymas Basin (21). Bipolaricaulota MAG-1503 also encodes an oxalate:formate antiporter distinct from those found in the *Methanosarcinaceae*, NPL-UPA2, and *Natrinincolaceae* MAGs (31-33% amino acid identities).

Thermodesulfovibrionales **MAG**

Two ASVs classified as *Thermodesulfovibrionia* (a class within phylum Nitrospirae) differed from each other by a single base. One of these was the top ASV in each of the fluid samples from Markers 3 and C (8-24% of all sequences), while the second ASV dominated the Calypso fluids (7-17% of all DNA fractions and 27% of the RNA fraction). Lost City *Thermodesulfovibrionia* metagenomic sequences and ASVs were notably rare in the low-sulfide fluids from Camel Humps and the high-sulfide fluids from Marker 2 (**Figures 2-3**).

Accordingly, a MAG (1293 CDS; 92% complete with 0% redundancy) classified as order *Thermodesulfovibrionales* within class *Thermodesulfovibrionia* was most abundant in Marker 3 and Calypso fluids. For heterotrophic metabolism, it encodes a nearly complete glycolysis pathway and TCA cycle, plus genes for lactate dehydrogenase, pyruvate ferredoxin oxidoreductase (PorABCD), and an oxalate:formate antiporter distinct from those found in the *Methanosarcinaceae*, NPL-UPA2, and *Natrinincolaceae* MAGs (21-24% amino acid identities),

Thermodesulfovibrionales MAG-1293 has a partial Wood-Ljungdahl pathway and a monomeric CO dehydrogenase (CooS) with two maturation factors (CooF and CooC). It encodes the NAD(P)-dependent formate dehydrogenase typical of acetogens (FdhA), as well as aerobic formate dehydrogenase (FdoG) and the divergent FDH-like sequence also observed in other Lost City MAGs (**Figure 6**). It encodes a [NiFe]-hydrogenase (HyaAB) classified as [NiFe] Group 1c, a group of respiratory H_2-uptake hydrogenases that can use fumarate, sulfate, or metals as terminal electron acceptors. The MAG contains only one subunit of membrane-bound hydrogenase (MbhJ), which shares 43-49% amino acid identities with the MbhJ sequences of the other Lost City MAGs shown in **Figure 5**. The key genes required for nitrogen fixation (nifHDK) and dissimilatory sulfate reduction (dsrAB) are present.

***Desulfotomaculum* MAGs**

Two MAGs classified as family *Desulfotomaculaceae* were resolved based on their distinct coverage patterns. Both MAGs were estimated to be 94% complete with 0% redundancy, but one had more predicted genes (1580) than the other (1144). *Desulfotomaculaceae* MAG-1580 was only abundant in one sample from Sombrero, while MAG-1144 was abundant in Marker 3, Sombrero, and Calypso fluids (**Figure 3**). Several ASVs were classified as genus *Desulfotomaculum* within the family *Desulfotomaculaceae*. One of these was most abundant in Marker 3 (up to 14% of all sequences) and Calypso (2-8% of all sequences), roughly matching

the distribution of MAG-1144, while another ASV (differing from the first by four bases) was only abundant in Sombrero fluids, similar to MAG-1580 (**Figure 2**).

Both *Desulfotomaculum* MAGs encode a complete or nearly complete glycolysis pathway and at least two genes of the TCA cycle (malate dehydrogenase and isocitrate dehydrogenase). In addition, *Desulfotomaculum* MAG-1144 has succinate dehydrogenase (SdhABC) and the beta subunit of fumarate dehydratase. *Desulfotomaculum* MAG-1580 encodes pyruvate ferredoxin oxidoreductase (PorABCD) and pyruvate formate-lyase (PflD). Both MAGs have a cation/acetate symporter (ActP), and MAG-1580 encodes a phosphonate transporter (PhnCDE).

Both MAGs have incomplete Wood-Ljungdahl pathways that lack the key enzyme CODH/ACS. Both MAGs also have monomeric CO dehydrogenase (CooS) and aerobic formate dehydrogenase (FdoG). No [NiFe] hydrogenases were detected, and only one subunit of [FeFe] hydrogenase (HndC) was present in the *Desulfotomaculum* MAGs. This hydrogenase is capable of H_2 oxidation with reduction of NADP in some organisms (22), but the presence of only one subunit in multiple Lost City MAGs (**Supplemental Table S5**) is curious and has unknown implications for the ability of these organisms to metabolize H_2.

The two *Desulfotomaculum* MAGs, in addition to the *Thermodesulfovibionales* MAG, are the only MAGs reported here that encode dissimilatory sulfite reductase (DsrAB). *Desulfotomaculum* dsrAB sequences were most abundant in Sombrero fluids, while *Thermodesulfovibrionales* dsrAB sequences were most abundant in Marker 3 and Calypso fluids. The two *Desulfotomaculum* MAGs also have the key genes required for nitrogen fixation (nifHDK).

***Natronincolaceae* MAGs**

Two MAGs were classified as family *Natronincolaceae* within the Clostridia and shared 81-89% ANI with a MAG reconstructed from seafloor borehole fluids at North Pond (23). Other genera within the family *Natronincolaceae* include *Alkaliphilus* and *Serpentinicella*, which have been isolated from the Prony Bay hydrothermal field (24, 25). The coverage of the *Natronincolaceae* MAGs was primarily in Sombrero fluids. The two MAGs shared 80% ANI but showed a few potentially important differences in their genomic inventories.

Natronincolaceae MAG-2163 (2163 CDS; 90% complete with 3% redundancy) has one of the largest genomes in this study, and it has an incomplete glycolysis pathway and incomplete TCA cycle. It encodes at least three steps of the Wood-Ljungdahl pathway, monomeric CODH (CooS), the Rnf complex, and the electron carriers HdrABC and MvhD.

In contrast, *Natronincolaceae* MAG-1138 (1138 CDS; 75% complete with 0% redundancy) has a smaller genome, a nearly complete glycolysis pathway, and no TCA cycle. It includes two genes associated with the Wood-Ljungdahl pathway, but not CODH, Rnf, Hdr, or MvhD. It does include acetate kinase (AckA) and phosphotransacetylase (Pta). It lacks any genes for ATP synthase, suggesting an obligate fermentative lifestyle.

Natronincolaceae MAG-2163 has [FeFe] hydrogenase (HndBCD), while MAG-1138 only has the HndC subunit. Both *Natronincolaceae* MAGs have at least one subunit of pyruvate dehydrogenase and pyruvate ferredoxin oxidoreductase, though they differ in which subunits they include (**Supplemental Table S5**).

***Dehalococcoidia* MAGs**

The most abundant *Dehalococcoidia* MAG (844 CDS; 73% complete with 0% redundancy) was classified as family SpSt-899 within order SZUA-161. It was one of the highest coverage MAGs

in Calypso fluids as well as the Sombrero and Marker 2 metatranscriptomes. Unlike the other two *Dehalococcoidia* MAGs described below, glycolysis and the TCA cycle are incomplete, and there are no genes required for the degradation of large organic compounds. However, the presence of pyruvate:ferredoxin oxidoreductase, pyruvate formate-lyase, and an oxalate/formate antiporter suggest the ability to ferment low-molecular-weight organic compounds. In addition, the MAG has two steps of the Wood-Ljungdahl pathway (FchA and MetF), the Rnf complex, and a complete set of genes encoding the key enzyme CODH/ACS. No hydrogenases or formate dehydrogenases are present. Curiously, *Dehalococcoidia* MAG-844 has V(A)-type ATP synthase, which is typically associated with archaea, but has also been observed in Chloroflexi and Parcubacteria in another serpentinite-hosted spring (26).

Two additional *Dehalococcoidia* MAGs belong to the SAR202 cluster, one of which was classified by GTDB as order SAR202 and one as order UBA3495. Each MAG has 98% ANI with previously published marine Chloroflexi MAGs (**Supplemental Table S4**). *Dehalococcoidia* MAG-2669 (2669 CDS; 86% complete with 4% redundancy) has moderately high coverage in all chimney fluids except for Marker 3. *Dehalococcoidia* MAG-2875 (2875 CDS; 87% complete with 1% redundancy) was less abundant than MAG-2669 in all samples but otherwise exhibited a similar distribution pattern. Both MAGs encoded nearly complete glycolysis pathways and TCA cycles, and they have a variety of genes associated with the oxidation of various organic molecules, consistent with previous studies of marine Chloroflexi (27, 28). Both MAGs encode cytochrome c oxidase (CoxA), which represents the only evidence for aerobic respiration in any of the final, refined MAGs. *Dehalococcoidia* MAG-2875 has both (aerobic) pyruvate dehydrogenase and (anaerobic) pyruvate:ferredoxin oxidoreductase, as reported for other SAR202 genomes (29), while MAG-2669 has neither. Neither MAG encodes any FDH or hydrogenases. MAG-2669 is predicted to encode sulfite reductase (Sir), adenylylsulfate reductase (AprAB), and sulfate adenylyltransferase (Sat), but dissimilatory sulfite reductase

(DsrAB) is not present. Both MAGs have genes associated with the metabolism of organosulfur compounds, similar to those reported by (29) for deep-sea SAR202 genomes (**Supplemental Table S5**).

All three *Dehalococcoidia* MAGs include genes for glycine reductase, thioredoxin, and selenocysteine synthesis. GrdB (beta subunit of glycine reductase) sequences from the two Lost City Dehalococcoidia MAGs that belong to the SAR202 marine cluster (MAG-2669 and MAG-2875) are distinct from the GrdB of *Dehalococcoidia* MAG-844 and from the GrdB of all other Lost City MAGs (**Supplemental Figure S11**).

NPL-UPA2 MAGs

Three MAGs classified as candidate phylum NPL-UPA2 (new name Candidatus Horikoshi bacteria proposed by (30) were recovered, each only 55-72% complete and with distinct coverage patterns among the hydrothermal fluid samples. No ASVs or unclassified reads were classified as NPL-UPA2, as this group was not yet represented in the SILVA or Kaiju databases. NPL-UPA2 MAG-914 (914 CDS; 62% complete with 0% redundancy) and MAG-1083 (1083 CDS; 55% complete with 3% redundancy) were most abundant in Calypso fluids and nearly absent in all other locations. MAG-718 (718 CDS; 72% complete with 3% redundancy) was most abundant in Marker 3 fluids and exhibited a nearly inverse abundance distribution compared to the other two MAGs. All three NPL-UPA2 MAGs have incomplete Wood-Ljungdahl pathways that lack the first few steps and begin with methylene-THF dehydrogenase (FolD), and all three MAGs encode V(A)-type ATP synthase and the Rnf complex, as reported by (30).

In most other respects the two NPL-UPA2 MAGs that are prominent in Calypso fluids differ from the MAG that is most abundant in Marker 3 fluids. The Calypso MAGs encode multiple subunits of the CODH/ACS enzyme, while MAG-718 has only the methyltransferase subunit (AcsE).

MAG-1083 also includes acetate kinase (AckA), phosphotransacetylase (Pta), and a cation/acetate symporter (ActP). In contrast, MAG-718 is the only NPL-UPA2 MAG that encodes pyruvate formate-lyase (PflD), the oxalate/formate antiporter, and carbonic anhydrase.

Evidence for hydrogenases in the NPL-UPA2 MAGs is lacking. Membrane-bound hydrogenase (MbhL) was identified in two of the MAGs by GhostKoala, but Prokka annotated these sequences as NAD(P)H-quinone oxidoreductase and formate hydrogenlyase. Neither sequence could be placed in the mbhL phylogeny of **Figure 5**. MAG-1083 includes one subunit of F_{420}-reducing hydrogenase (FrhB) and one subunit of [FeFe] hydrogenase (HndD), but the functional roles of these predicted proteins are unclear.

Unlike the NPL-UPA2 MAG reported by (30), none of the Lost City NPL-UPA2 MAGs have formate dehydrogenase nor the electron carriers Hdr and Etf.

Patescibacteria MAGs

Several MAGs classified as candidate phylum Patescibacteria were represented by two classes: Paceibacteria and Gracilibacteria (according to GTDB taxonomy). Paceibacteria MAGs (55-86% completion with 0-3% redundancy) had small genomes (as low as 307 kb with 63% estimated completion) with low GC content (24-45%). Gracilibacteria MAGs (61-83% completion with 0-1% redundancy) had even lower GC content (22-34%) and somewhat larger genomes but with very low annotation success. As few as 8% of all coding sequences in Gracilibacteria MAGs yielded GhostKoala results. Two of the Paceibacteria MAGs were most abundant in Marker 3 fluids, while Gracilibacteria MAGs were notably absent in Marker 3 fluids and were more abundant in Camel Humps and Sombrero fluids (**Figure 3; Supplemental Table S4**).

In general, Paceibacteria and Gracilibacteria MAGs included genes for the biosynthesis of key cellular components and basic information processing, but genes specific to catabolic pathways were rare. The few exceptions included a lactate transporter (LctP) that was present in the two highest-coverage Paceibacteria MAGs, and one of these MAGs also has lactate dehydrogenase (LdhA). In addition, acetate kinase (AckA), acylphosphatase (AcyP), and phosphoenolpyruvate synthase (PpsA) were present in one or more Paceibacteria MAGs (**Supplemental Table S5**). Propionyl-CoA carboxylase (PccB) was included in one Gracilibacteria MAG. All Paceibacteria MAGs, one of the Gracilibacteria MAGs, and almost all other MAGs in this study have a substrate-binding protein associated with the peptide/nickel transport system (K02035; **Supplemental Table S6**).

Curiously, methylene tetrahydrofolate dehydrogenase (FolD), which is part of the Wood-Ljungdahl pathway, was encoded by three Paceibacteria MAGs and one Gracilibacteria MAG. FolD was also observed in 17 MAGs from The Cedars, a serpentinite-hosted spring in California, that were classified as candidate phylum OD1 (26), now included within Paceibacteria in GTDB.

As mentioned in the main text, ATP synthase was completely absent in three of the Paceibacteria MAGs, and one Gracilibacteria MAG included only a single subunit. One Paceibacteria MAG encodes a V(A)-type ATP synthase instead of the F-type ATP synthase present in the other Paceibacteria and Gracilibacteria MAGs. MAGs that were classified as candidate phylum OD1 from The Cedars also encoded V(A)-type ATP synthase or lacked any ATP synthase at all (26).

WOR-3 MAG

One of the highest-coverage MAGs in Marker 3 and Sombrero fluids was classified as candidate phylum WOR-3, which was previously identified in methane-rich marine sediments (31) and was proposed to be renamed as Candidatus Stahlbacteria (32) The WOR-3 MAG (59% completion with 0% redundancy) has a partial glycolysis pathway, perhaps explainable by the incompleteness of the MAG, and no TCA cycle. Other genes possibly indicative of organic carbon catabolism include those predicted to encode one subunit of pyruvate dehydrogenase (PdhD), pyruvate formate-lyase (PflD), formate dehydrogenase (FdoG), and a cation/acetate symporter (ActP). Like the NPL-UPA2 MAGs, one Paceibacteria MAG, and the archaeal MAGs, the WOR-3 MAG encodes a V(A)-type ATP synthase.

The WOR-3 formate dehydrogenase shares a maximum of only ~31% amino acid identities with any other sequences in the NCBI NR and JGI IMG “all isolates” databases. This divergent FDH-like sequence in the WOR-3 MAG is distinct from the divergent FDH-like sequence described above for other Lost City MAGs (**Figure 6**), and in both cases, additional research is required to establish whether their functions are indeed associated with formate metabolism.

References

1. Brazelton WJ, Schrenk MO, Kelley DS, Baross JA. 2006. Methane- and Sulfur-Metabolizing Microbial Communities Dominate the Lost City Hydrothermal Field Ecosystem. Appl Environ Microbiol 72:6257–6270.

2. Schrenk MO, Kelley DS, Bolton SA, Baross JA. 2004. Low archaeal diversity linked to subseafloor geochemical processes at the Lost City Hydrothermal Field, Mid-Atlantic Ridge. Environ Microbiol 6:1086–1095.

3. Brazelton WJ, Ludwig KA, Sogin ML, Andreishcheva EN, Kelley DS, Shen C-C, Edwards RL, Baross JA. 2010. Archaea and bacteria with surprising microdiversity show shifts in

dominance over 1,000-year time scales in hydrothermal chimneys. Proceedings of the National Academy of Sciences 107:1612–1617.

4. Brazelton WJ, Mehta MP, Kelley DS, Baross JA. 2011. Physiological Differentiation within a Single-Species Biofilm Fueled by Serpentinization. mBio 2:e00127-11.

5. Koch H, Germscheid N, Freese HM, Noriega-Ortega B, Lücking D, Berger M, Qiu G, Marzinelli EM, Campbell AH, Steinberg PD, Overmann J, Dittmar T, Simon M, Wietz M. 2020. Genomic, metabolic and phenotypic variability shapes ecological differentiation and intraspecies interactions of Alteromonas macleodii. Sci Rep 10:809.

6. Brazelton WJ, Baross JA. 2010. Metagenomic Comparison of Two Thiomicrospira Lineages Inhabiting Contrasting Deep-Sea Hydrothermal Environments. PLoS ONE 5:e13530.

7. McGonigle JM, Lang SQ, Brazelton WJ. 2020. Genomic Evidence for Formate Metabolism by *Chloroflexi* as the Key to Unlocking Deep Carbon in Lost City Microbial Ecosystems. Appl Environ Microbiol 86:e02583-19.

8. Brazelton WJ, Baross JA. 2009. Abundant transposases encoded by the metagenome of a hydrothermal chimney biofilm. ISME J 3:1420–1424.

9. Mand TD, Metcalf WW. 2019. Energy Conservation and Hydrogenase Function in Methanogenic Archaea, in Particular the Genus Methanosarcina. Microbiol Mol Biol Rev 83.

10. Thauer RK, Kaster A-K, Goenrich M, Schick M, Hiromoto T, Shima S. 2010. Hydrogenases from Methanogenic Archaea, Nickel, a Novel Cofactor, and H_2 Storage. Annu Rev Biochem 79:507–536.

11. Lang SQ, Früh-Green GL, Bernasconi SM, Brazelton WJ, Schrenk MO, McGonigle JM. 2018. Deeply-sourced formate fuels sulfate reducers but not methanogens at Lost City hydrothermal field. Sci Rep 8:755.

12. Abe K, Ruan Z-S, Maloney PC. 1996. Cloning, Sequencing, and Expression in Escherichia coli of OxlT, the Oxalate:Formate Exchange Protein of Oxalobacter formigenes(∗). Journal of Biological Chemistry 271:6789–6793.

13. Fones EM, Colman DR, Kraus EA, Stepanauskas R, Templeton AS, Spear JR, Boyd ES. 2021. Diversification of methanogens into hyperalkaline serpentinizing environments through adaptations to minimize oxidant limitation. ISME J 15:1121–1135.

14. Biegel E, Schmidt S, González JM, Müller V. 2011. Biochemistry, evolution and physiological function of the Rnf complex, a novel ion-motive electron transport complex in prokaryotes. Cell Mol Life Sci 68:613–634.

15. Chadwick GL, Skennerton CT, Laso-Pérez R, Leu AO, Speth DR, Yu H, Morgan-Lang C, Hatzenpichler R, Goudeau D, Malmstrom R, Brazelton WJ, Woyke T, Hallam SJ, Tyson GW, Wegener G, Boetius A, Orphan VJ. 2022. Comparative genomics reveals electron transfer and syntrophic mechanisms differentiating methanotrophic and methanogenic archaea. PLOS Biology 20:e3001508.

16. Beulig F, Røy H, McGlynn SE, Jørgensen BB. 2019. Cryptic CH4 cycling in the sulfate–methane transition of marine sediments apparently mediated by ANME-1 archaea. ISME J 13:250–262.

17. Parks DH, Chuvochina M, Rinke C, Mussig AJ, Chaumeil P-A, Hugenholtz P. 2021. GTDB: an ongoing census of bacterial and archaeal diversity through a phylogenetically

consistent, rank normalized and complete genome-based taxonomy. Nucleic Acids Res https://doi.org/10.1093/nar/gkab776.

18. Nobu MK, Nakai R, Tamazawa S, Mori H, Toyoda A, Ijiri A, Suzuki S, Kurokawa K, Kamagata Y, Tamaki H. 2021. Unique metabolic strategies in Hadean analogues reveal hints for primordial physiology.

19. Brazelton WJ, Thornton CN, Hyer A, Twing KI, Longino AA, Lang SQ, Lilley MD, Früh-Green GL, Schrenk MO. 2017. Metagenomic identification of active methanogens and methanotrophs in serpentinite springs of the Voltri Massif, Italy. PeerJ 5:e2945.

20. Zhou Z, Liu Y, Pan J, Cron BR, Toner BM, Anantharaman K, Breier JA, Dick GJ, Li M. 2020. Gammaproteobacteria mediating utilization of methyl-, sulfur- and petroleum organic compounds in deep ocean hydrothermal plumes. ISME J 14:3136–3148.

21. Dombrowski N, Teske AP, Baker BJ. 2018. Expansive microbial metabolic versatility and biodiversity in dynamic Guaymas Basin hydrothermal sediments. Nat Commun 9:4999.

22. Kpebe A, Benvenuti M, Guendon C, Rebai A, Fernandez V, Le Laz S, Etienne E, Guigliarelli B, García-Molina G, de Lacey AL, Baffert C, Brugna M. 2018. A new mechanistic model for an O2-protected electron-bifurcating hydrogenase, Hnd from Desulfovibrio fructosovorans. Biochimica et Biophysica Acta (BBA) - Bioenergetics 1859:1302–1312.

23. Tully BJ, Wheat CG, Glazer BT, Huber JA. 2018. A dynamic microbial community with high functional redundancy inhabits the cold, oxic subseafloor aquifer. ISME J 12:1–16.

24. Mei N, Postec A, Erauso G, Joseph M, Pelletier B, Payri C, Ollivier B, Quéméneur M. 2016. Serpentinicella alkaliphila gen. nov., sp. nov., a novel alkaliphilic anaerobic

bacterium isolated from the serpentinite-hosted Prony hydrothermal field, New Caledonia. Int J Syst Evol Microbiol 66:4464–4470.

25. Postec A, Quéméneur M, Lecoeuvre A, Chabert N, Joseph M, Erauso G. 2021. Alkaliphilus serpentinus sp. nov. and Alkaliphilus pronyensis sp. nov., two novel anaerobic alkaliphilic species isolated from the serpentinite-hosted Prony Bay Hydrothermal Field (New Caledonia). Systematic and Applied Microbiology 44:126175.

26. Suzuki S, Ishii S, Hoshino T, Rietze A, Tenney A, Morrill PL, Inagaki F, Kuenen JG, Nealson KH. 2017. Unusual metabolic diversity of hyperalkaliphilic microbial communities associated with subterranean serpentinization at The Cedars. ISME J 11:2584–2598.

27. Liu R, Wei X, Wang L, Cao J, Song W, Wu J, Thomas T, Jin T, Wang Z, Wei W, Wei Y, Zhai H, Yao C, Shen Z, Fang J. 2021. Novel Chloroflexi Genomes From The Deepest Ocean Reveal Metabolic Strategies For The Adaptation To Deep-Sea Habitats https://doi.org/10.21203/rs.3.rs-254541/v2.

28. Landry Z, Swan BK, Herndl GJ, Stepanauskas R, Giovannoni SJ. SAR202 Genomes from the Dark Ocean Predict Pathways for the Oxidation of Recalcitrant Dissolved Organic Matter. mBio 8:e00413-17.

29. Mehrshad M, Rodriguez-Valera F, Amoozegar MA, López-García P, Ghai R. 2018. The enigmatic SAR202 cluster up close: shedding light on a globally distributed dark ocean lineage involved in sulfur cycling. ISME J 12:655–668.

30. Suzuki S, Nealson KH, Ishii S. 2018. Genomic and in-situ Transcriptomic Characterization of the Candidate Phylum NPL-UPL2 From Highly Alkaline Highly Reducing Serpentinized Groundwater. Frontiers in Microbiology 9:3141.

31. Baker BJ, Lazar CS, Teske AP, Dick GJ. 2015. Genomic resolution of linkages in carbon, nitrogen, and sulfur cycling among widespread estuary sediment bacteria. Microbiome 3:14.

32. Dombrowski N, Seitz KW, Teske AP, Baker BJ. 2017. Genomic insights into potential interdependencies in microbial hydrocarbon and nutrient cycling in hydrothermal sediments. Microbiome 5:106.

Tables and Figures
Brazelton et al. “Metabolic strategies shared by basement residents of the Lost City hydrothermal field”

Tables
1. Sample overview including temp and chemistry

Figures
1. Map and ordination
2. 16S bubbles
3. MAG bubbles
4. MAG presence/absence of key genes
5. MBH phylogeny
6. FDH phylogeny
7. Key gene bubbles

Supplemental Figures
1. Extended map figure
2. Chimney photos
3. Metagenome analysis workflow
4. Kaiju bubbles
5. Phylogeny – Methanosarcinales 16S + mcrA
6. Phylogeny – Bipolaricaulota 16S
7. Phylogeny – Thermodesulfovibrionales 16S
8. Phylogeny + gene order mbhL
9. Phylogeny + gene order FDH
10. Phylogeny – carbonic anhydrase
11. Phylogeny – GrdB
12. Hydrogenase bubbles
13. Acetate/formate bubbles
14. Methanogenesis bubbles
15. Acetogenesis bubbles

Supplemental Tables (Excel files)
1. Sample info
2. Full 16S count table including contaminants
3. Kaiju tables
4. MAG taxonomy, completeness, coverage table
5. MAG gene presence absence tables
6. MAG annotations for transporters, dbCAN, FeGenie
7. KO coverage table
8. Incubation experiment results

Github Repo
1. Protocols
2. Kaiju Krona plots
3. NCBI SRA and GenBank metadata
4. MAG sequences and annotations
5. Alignments and sequences for phylogenetic trees
6. R code for plots
7. Python scripts for metagenomic analyses

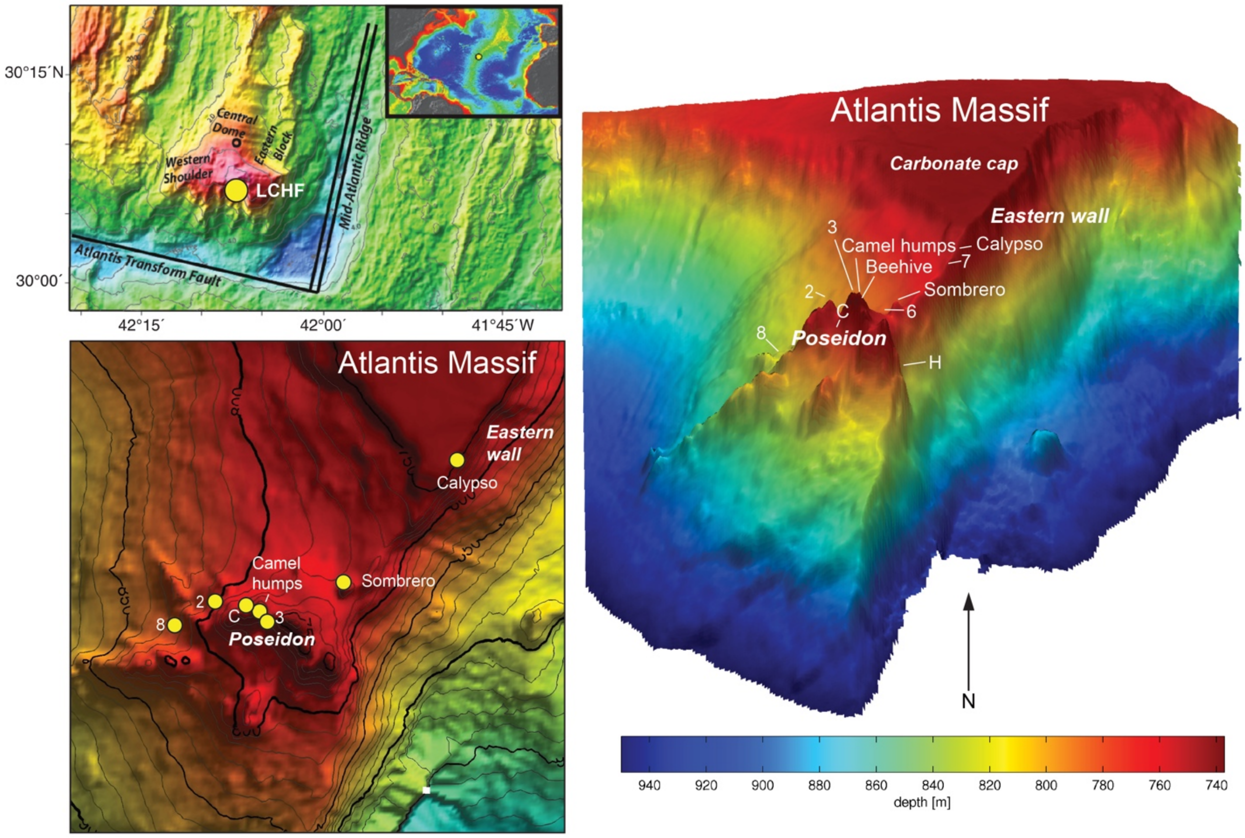

Supplemental Figure S1. Extended version of **Figure 1** showing the location of the Lost City hydrothermal field near the summit of the Atlantis Massif, which is located northwest of the intersection of the Mid-Atlantic Ridge and the Atlantis Transform Fault.

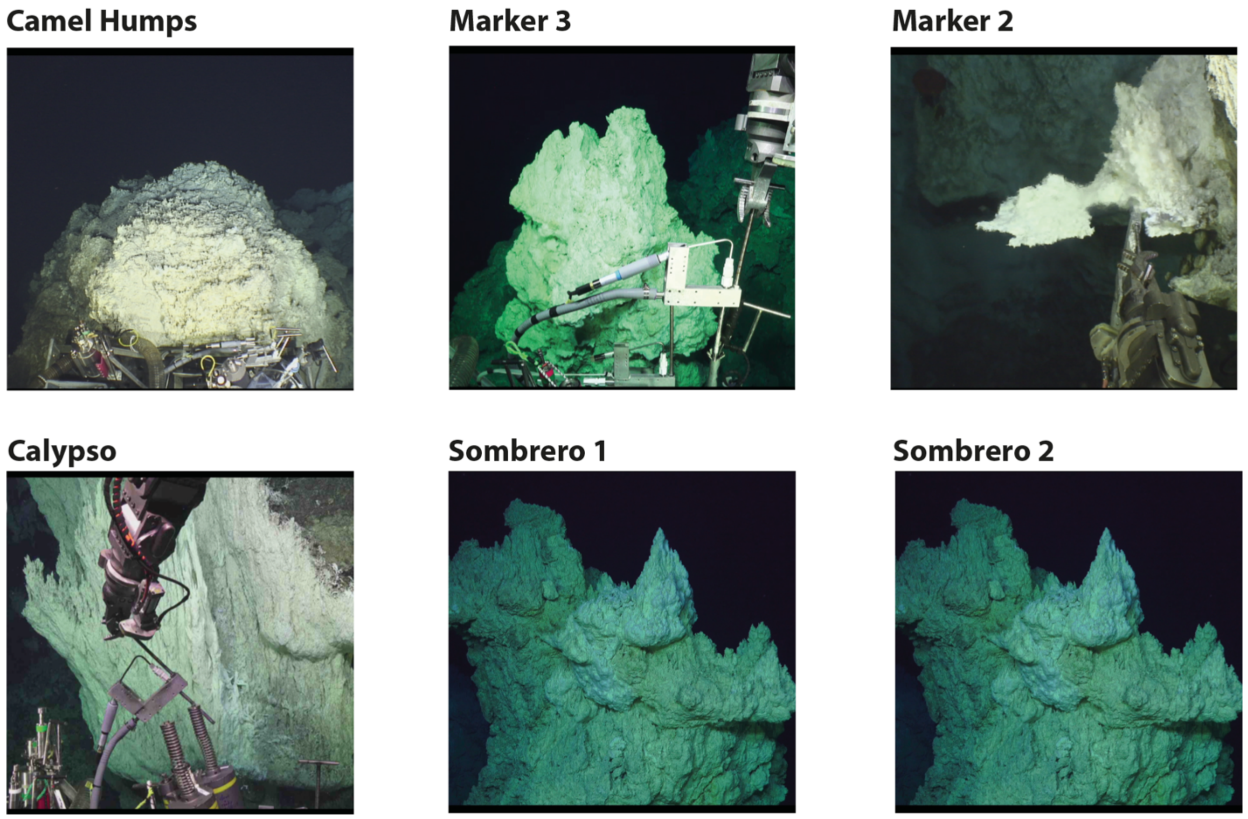

Supplemental Figure S2. Photographs of sampling locations for this study, captured on the seafloor by ROV *Jason*. Camel Humps and Marker 3 are visually distinct structures despite their nearby locations. Sombrero was sampled at the same location on two separate dives.

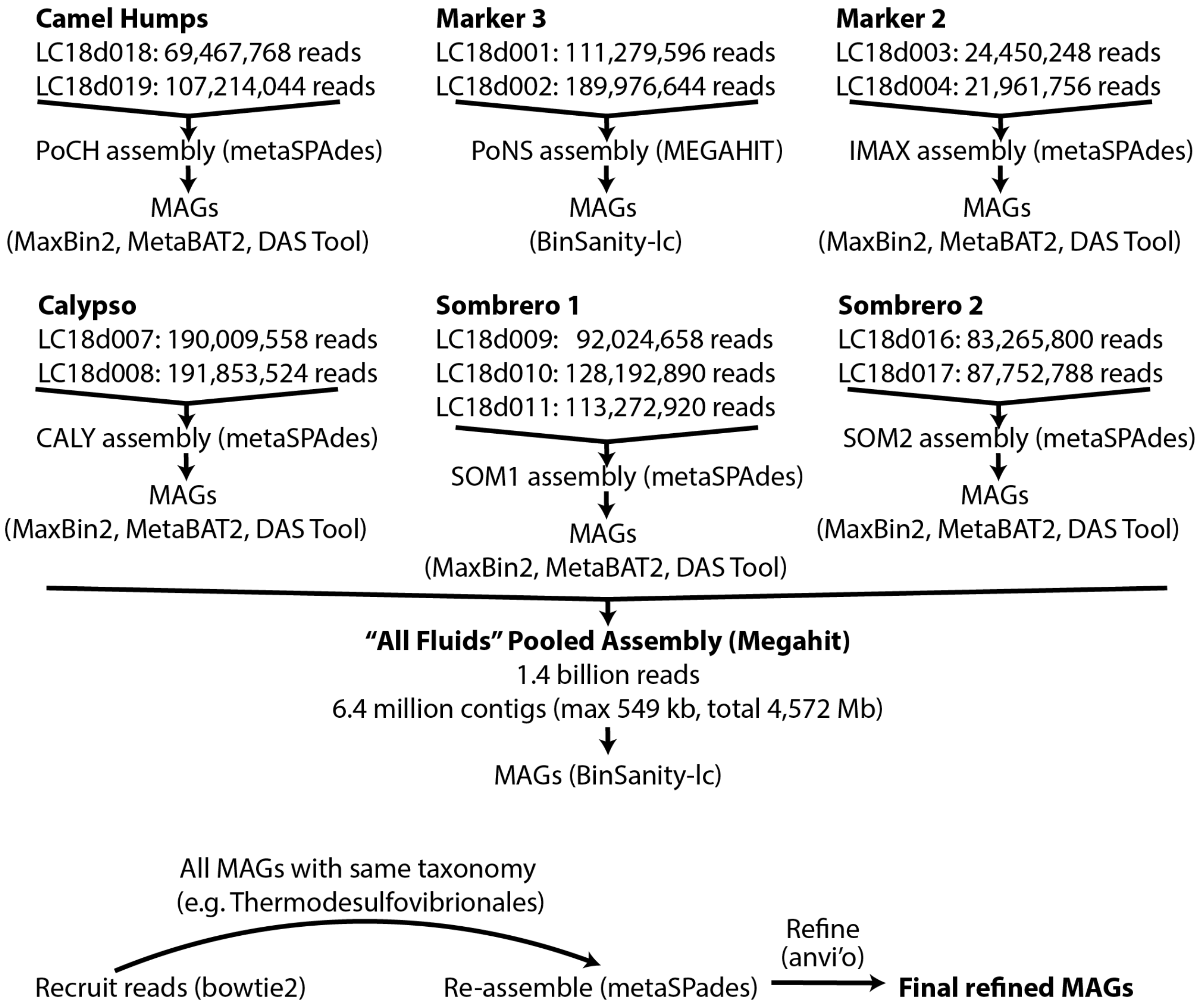

Supplemental Figure S3. Overall workflow for assembly of metagenomes and binning into metagenome-assembled genomes (MAGs). Assemblies were performed with reads pooled from each chimney location (chimney-specific assemblies), and one "all fluids" pooled assembly was performed with all metagenomic reads from all chimney locations. Initial bins constructed with automated tools were used as a template for recruiting metagenomic reads for a re-assembly and manual curation and refinement of the final MAGs.

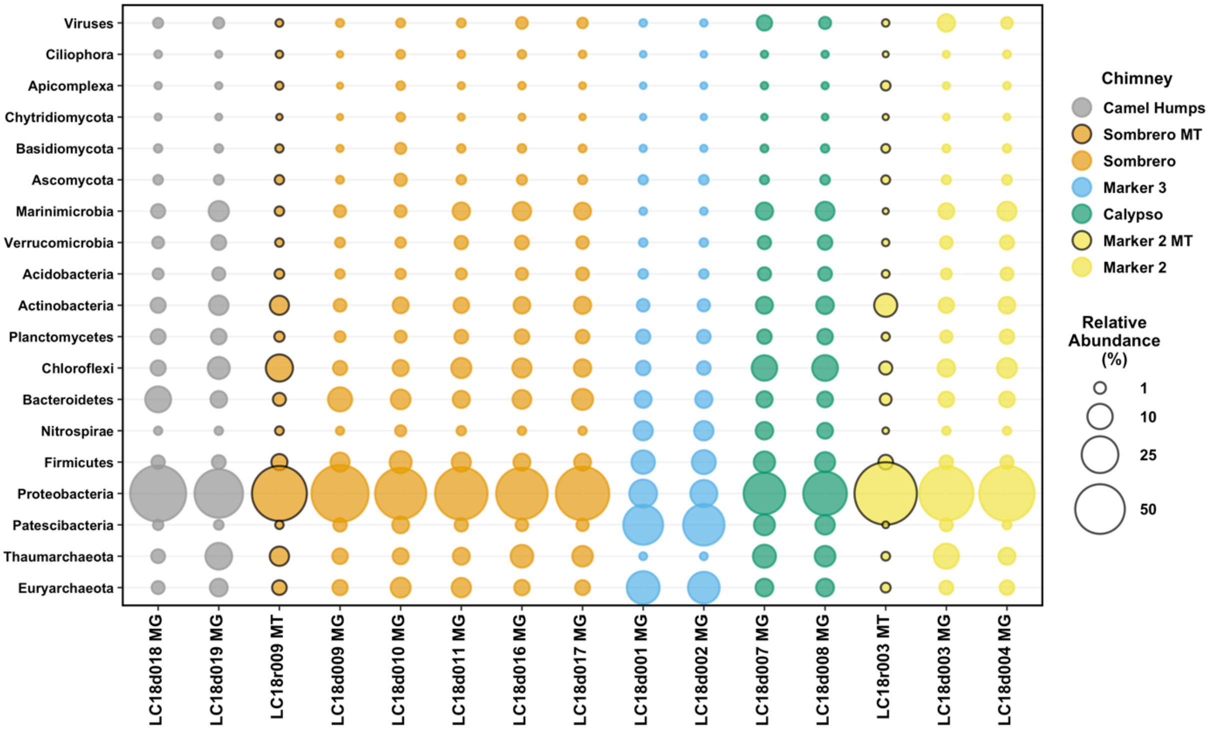

Supplemental Figure S4. Percent of reads classified to the top 18 phyla (plus viruses) in Lost City hydrothermal fluid samples. Unassembled reads were classified using Kaiju with its default NCBI nr+euk database. Percentages were calculated as the number of reads classified to each phyla divided by the total number of reads in that library that could be classified to the phylum level by Kaiju. Bubbles representing reads in metatranscriptomes (MT), rather than metagenomes (MG), are highlighted with black borders.

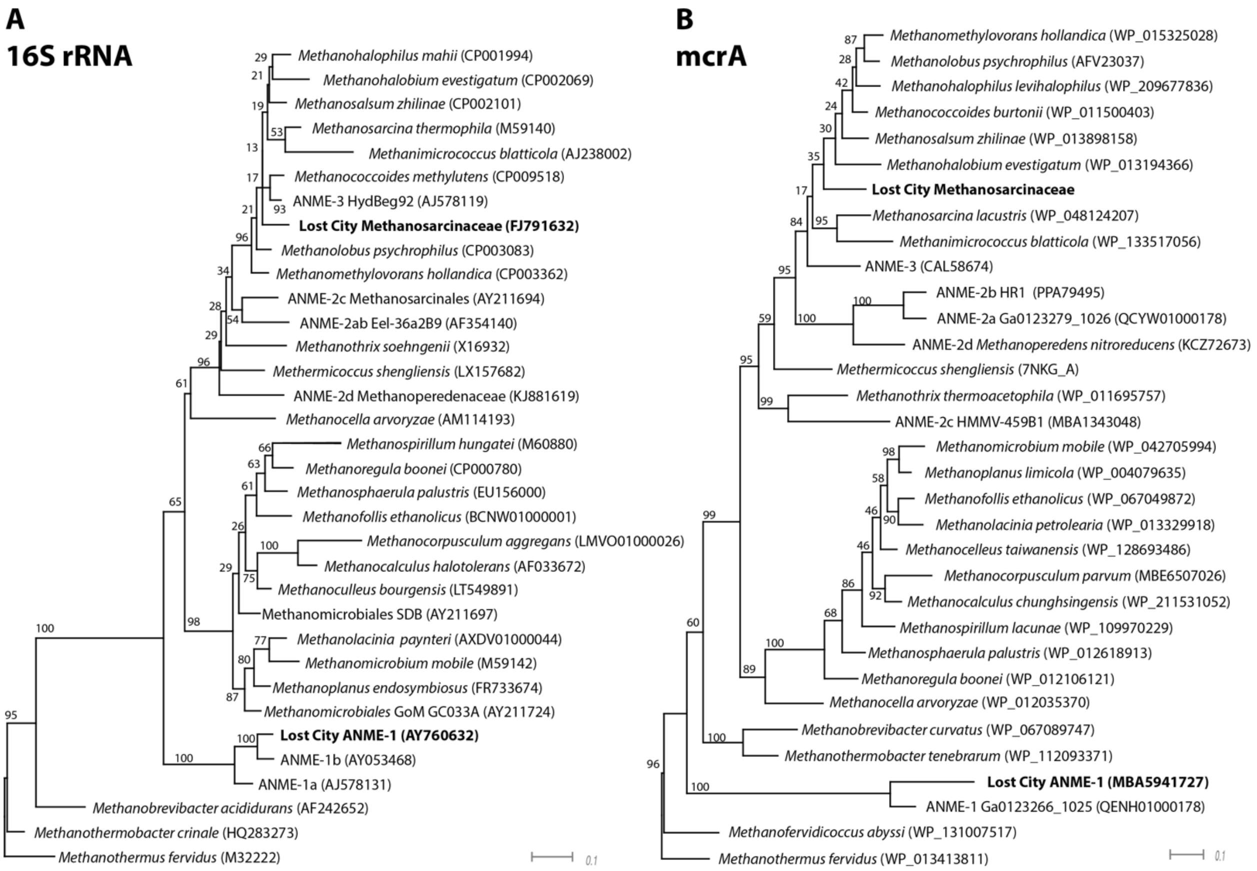

Supplemental Figure S5. Phylogenies of 16S rRNA and mcrA (alpha subunit of methyl coenzyme M reductase) highlighting Lost City MAGs classified as Methanosarcinaceae and ANME-1. Bootstrap support values are shown for each node. Sequences and accession IDs are provided in the Zenodo-archived GitHub repository accessible via DOI: 10.5281/zenodo.5798015.

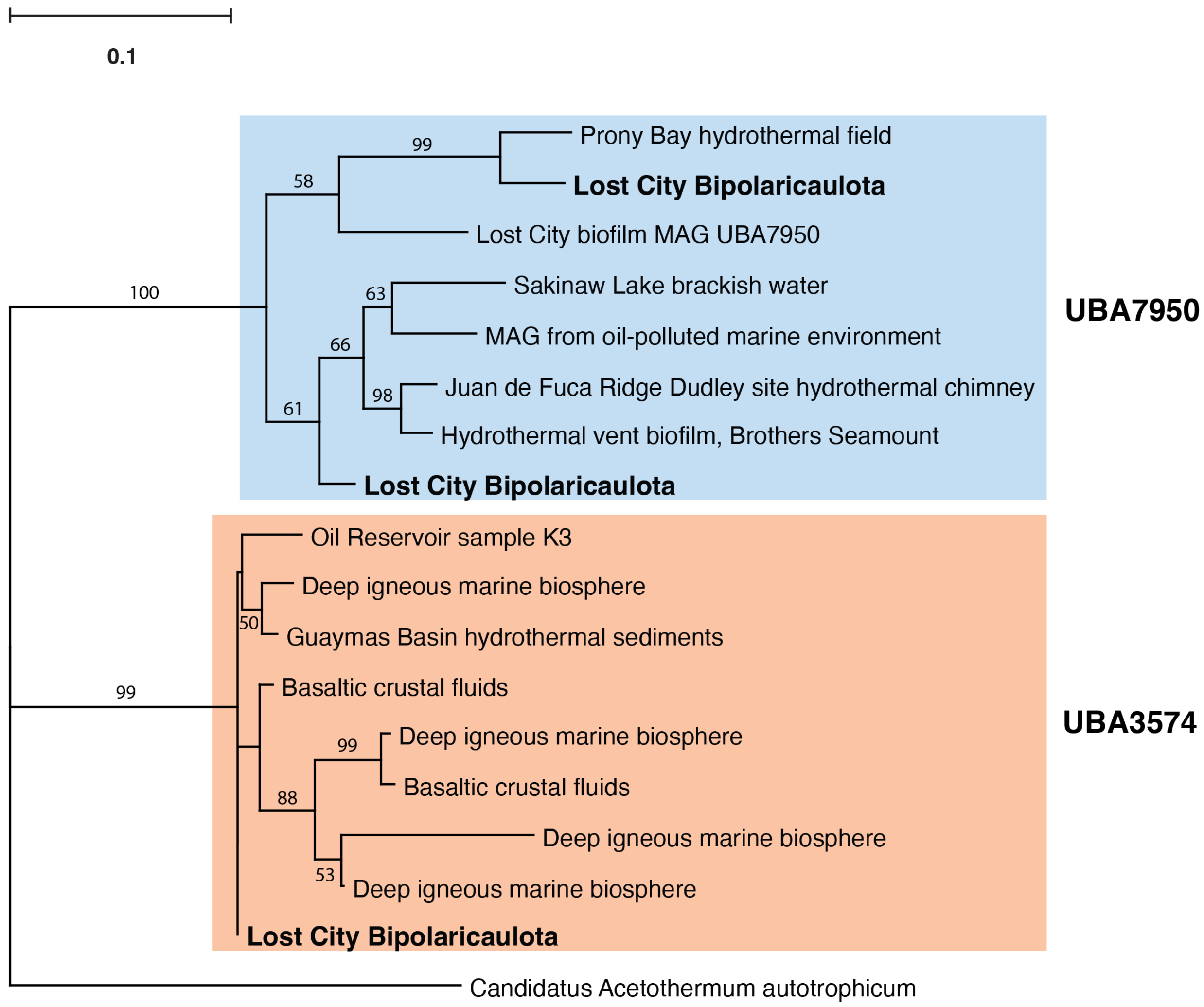

Supplemental Figure S6. Phylogeny of 16S rRNA highlighting Lost City sequences classified as Bipolaricaulota. The most abundant Lost City Bipolaricaulota 16S rRNA sequences cluster into two distinct monophyletic groups, classified by GTDB as UBA3574 and UBA7950, which corresponds to the classifications of the three refined Bipolaricaulota MAGs (**Figure 3**). The UBA7950 sequences are further divided into two clades, one of which includes a MAG assembled by Parks et al. (2018) from our previous study of Lost City chimney biofilms (DOHL01000117). Bootstrap support values greater than 50 are shown for each node. Sequences and accession IDs are provided in the Zenodo-archived GitHub repository accessible via DOI: 10.5281/zenodo.5798015.

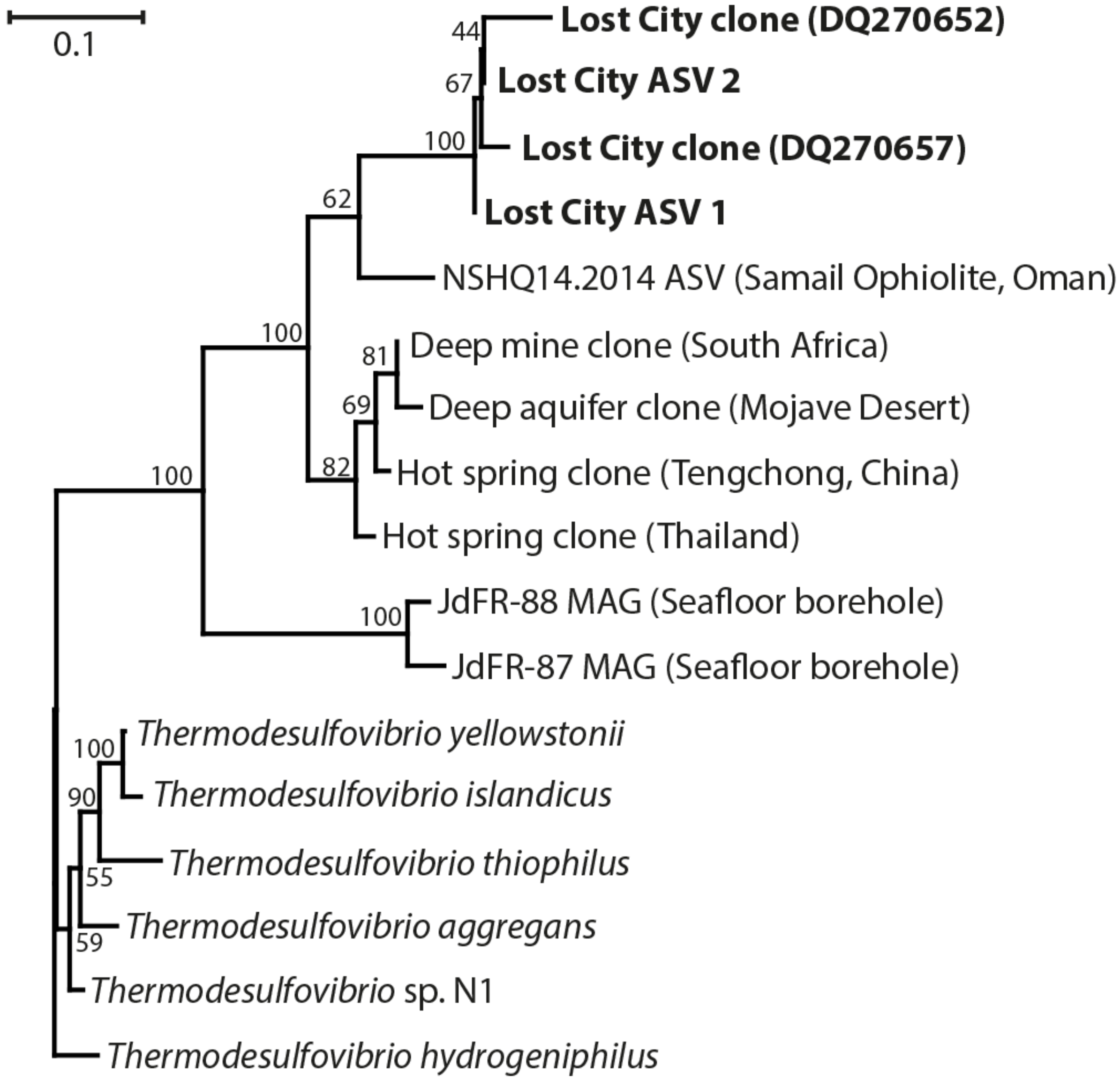

Supplemental Figure S7. Phylogeny of 16S rRNA highlighting Lost City sequences classified as Thermodesulfovibrionia. The two Lost City ASVs differ from each other by a single base and match sequences from a previously published clone library of Lost City chimney biofilms (Brazelton et al., 2006). They share 90% nucleotide identities with their closest neighbor, an ASV from alkaline borehole fluids in the Samail Ophiolite, Oman (Rempfert et al., 2017). Sequences and accession IDs are provided in the Zenodo-archived GitHub repository accessible via DOI: 10.5281/zenodo.5798015.

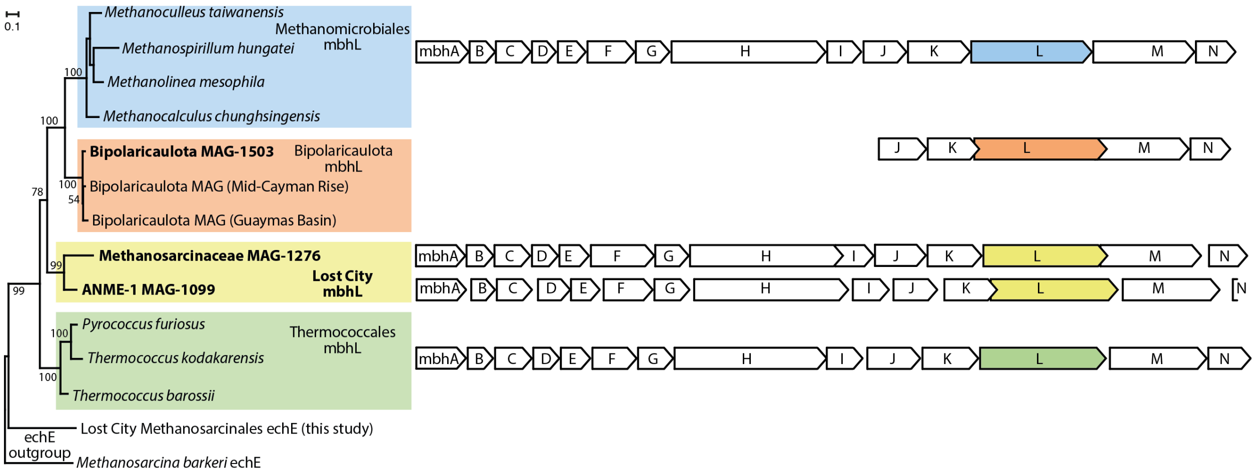

Supplemental Figure S8. Phylogeny of the large catalytic subunit of membrane-bound hydrogenase (mbhL) and the mbh gene cluster (expanded version of Figure 5). Sequences and accession IDs are provided in the Zenodo-archived GitHub repository accessible via DOI: 10.5281/zenodo.5798015.

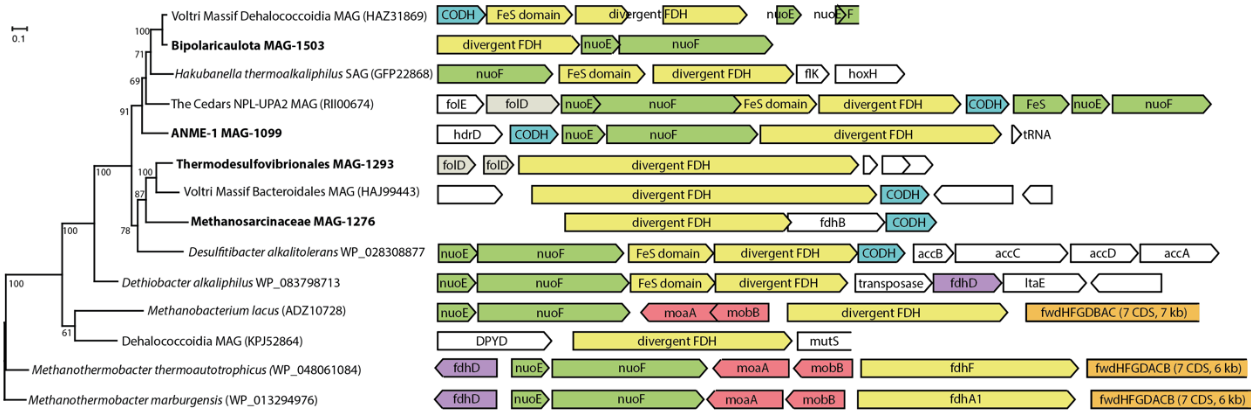

Supplemental Figure S9. Phylogeny of divergent FDH-like sequences (expanded version of Figure 6). In most cases, the divergent FDH-like gene was flanked by nuoEF (encoding NADH-quinone oxidoreductase) and a hypothetical sequence with a conserved domain associated with monomeric carbon monoxide dehydrogenase (CODH). Furthermore, most of these gene clusters contained signs of genome instability just upstream or downstream such as pseudogenes, transposases, or a toxin/antitoxin system (not shown here). Sequences and accession IDs are provided in the Zenodo-archived GitHub repository accessible via DOI: 10.5281/zenodo.5798015.

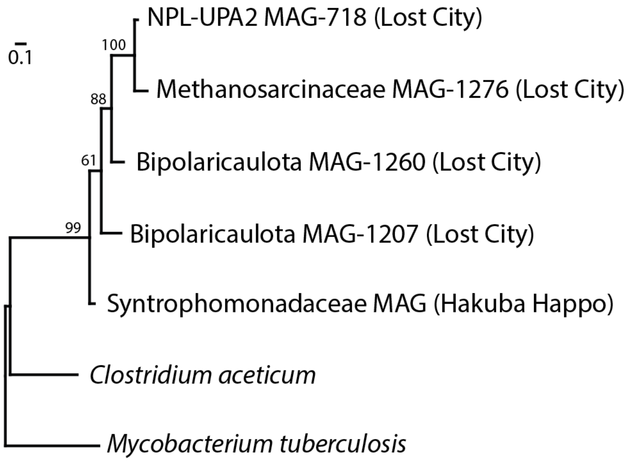

Supplemental Figure S10. Phylogeny of divergent sequences predicted to encode carbonic anhydrase. Lost City sequences form a novel clade including a predicted sequence from a MAG recovered from another serpentinite-hosted spring (Hakuba Happo). Bootstrap support values are shown for each node. Sequences and accession IDs are provided in the Zenodo-archived GitHub repository accessible via DOI: 10.5281/zenodo.5798015.

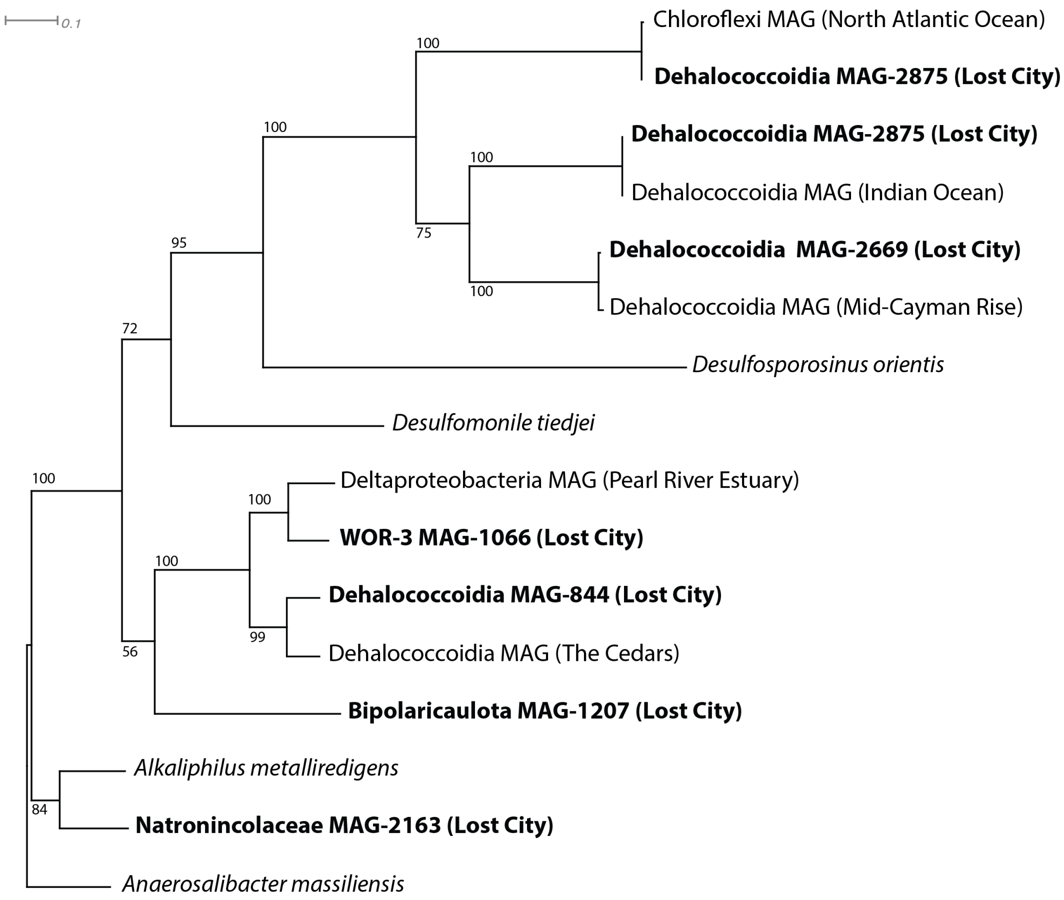

Supplemental Figure S11. Phylogeny of GrdB (beta subunit of glycine reductase). Lost City Bipolaricaulota, Dehalococcoidia, WOR-3, and Natronincolaceae MAGs share moderate sequence similarity (58-88% amino acid identities) with sequences from other MAGs (including one from another site of serpentinization, The Cedars), but limited similarity with sequences from characterized species. Lost City Dehalococcoidia MAGs that belong to the SAR202 marine cluster, including two copies from MAG-2875, form a separate clade from other Lost City MAGs that are more likely to represent subseafloor organisms. A second Natronincolaceae MAG not shown here lacks GrdB but includes all other genes associated with glycine reductase (**Supplemental Table S5**). Bootstrap support values are shown for each node. Sequences and accession IDs are provided in the Zenodo-archived GitHub repository accessible via DOI: 10.5281/zenodo.5798015.

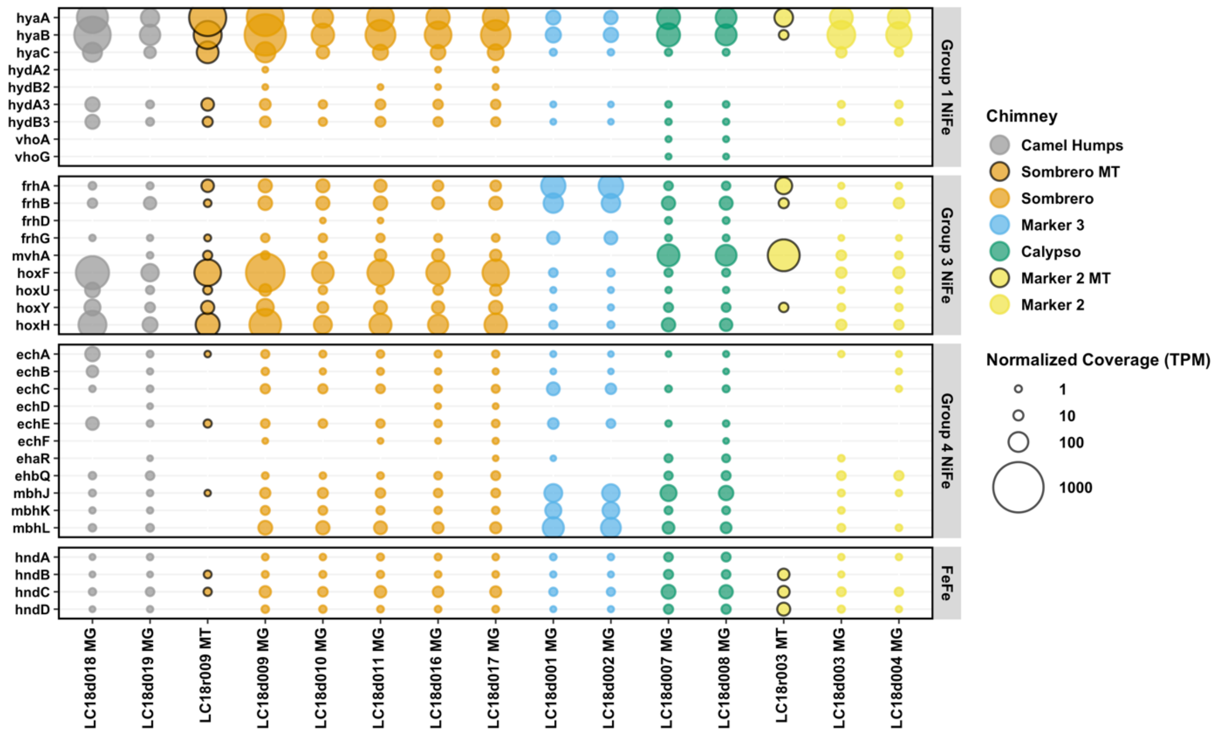

Supplemental Figure S12. Abundance of predicted hydrogenase sequences in Lost City hydrothermal fluid samples. Metagenomic coverage was normalized to predicted protein length and to the size of the metagenome or metatranscriptome library. The final normalized coverage is reported as a proportional unit (transcripts/fragments per million; TPM) suitable for cross-sample comparisons. Bubbles representing coverage in metatranscriptomes (MT), rather than metagenomes (MG), are highlighted with black borders. Genes are defined with KEGG Orthology; see **Supplemental Table S5**.

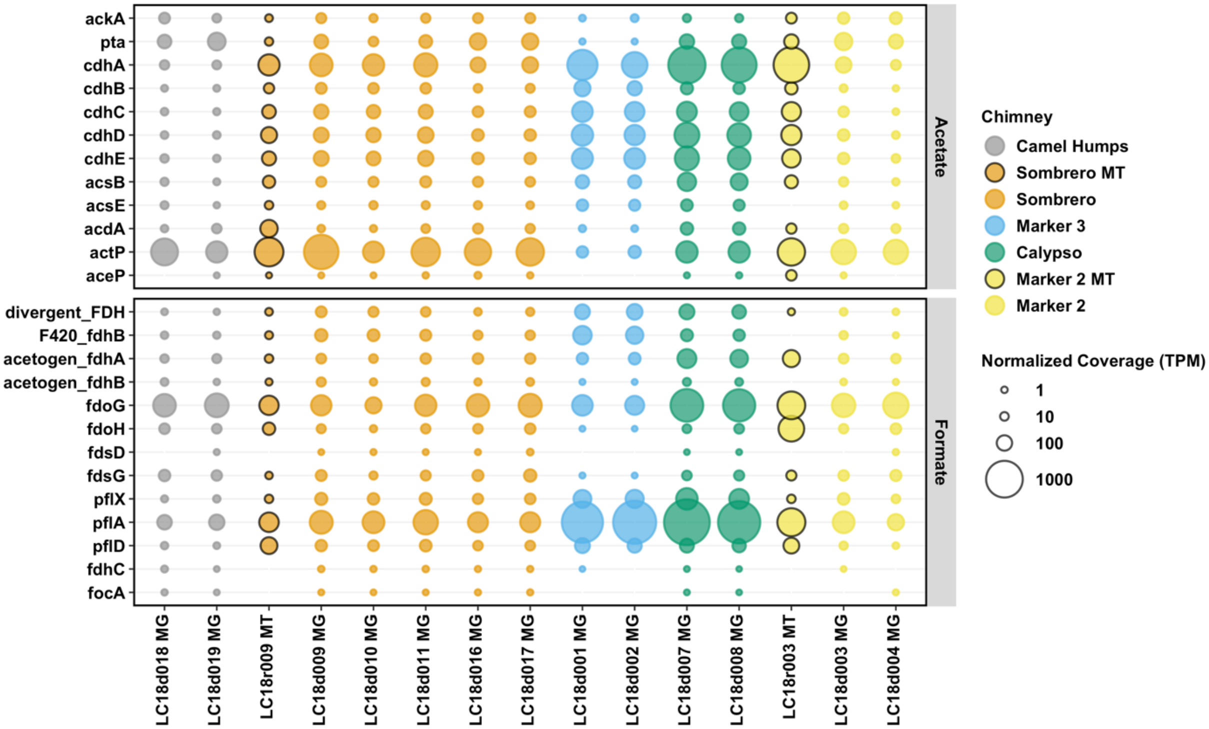

Supplemental Figure S13. Abundances of predicted sequences associated with acetate and formate metabolism in Lost City hydrothermal fluid samples. Metagenomic coverage was normalized to predicted protein length and to the size of the metagenome or metatranscriptome library. The final normalized coverage is reported as a proportional unit (transcripts/fragments per million; TPM) suitable for cross-sample comparisons. Bubbles representing coverage in metatranscriptomes (MT), rather than metagenomes (MG), are highlighted with black borders. Genes are defined with KEGG Orthology; see **Supplemental Table S5**.

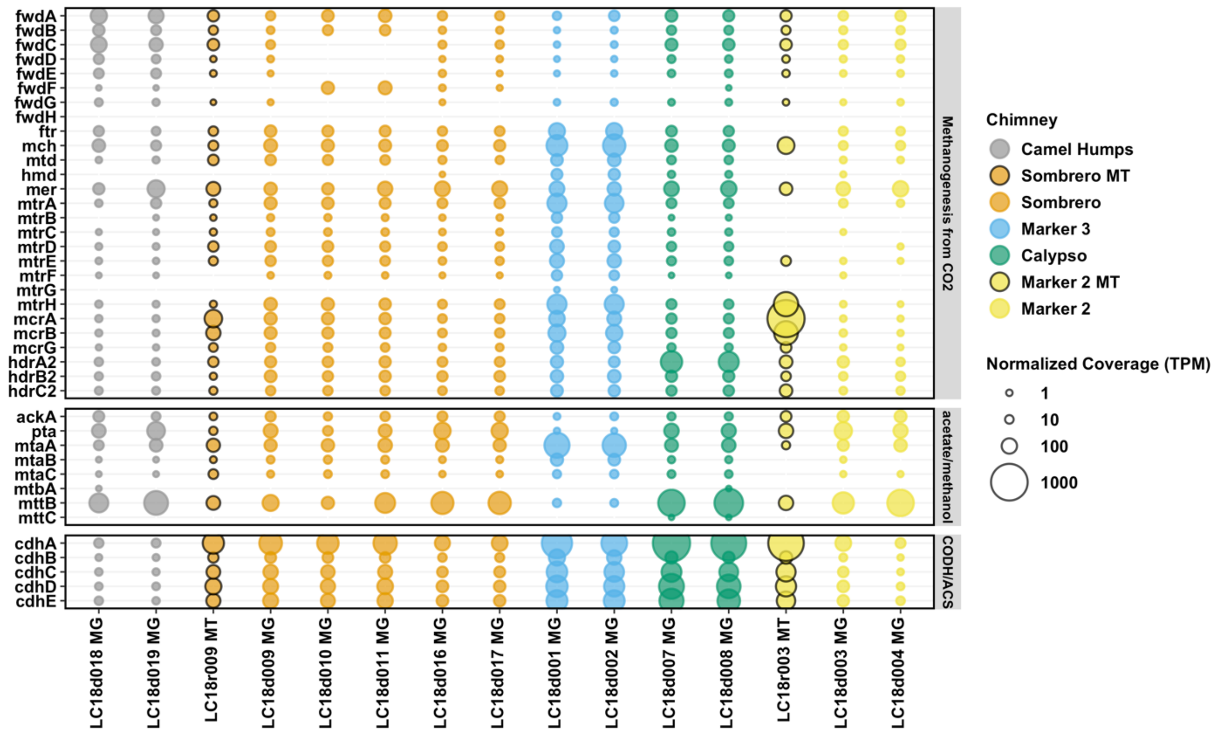

Supplemental Figure S14. Abundance of predicted sequences associated with methanogenesis in Lost City hydrothermal fluid samples. Metagenomic coverage was normalized to predicted protein length and to the size of the metagenome or metatranscriptome library. The final normalized coverage is reported as a proportional unit (transcripts/fragments per million; TPM) suitable for cross-sample comparisons. Bubbles representing coverage in metatranscriptomes (MT), rather than metagenomes (MG), are highlighted with black borders. Genes are defined with KEGG Orthology; see **Supplemental Table S5**.

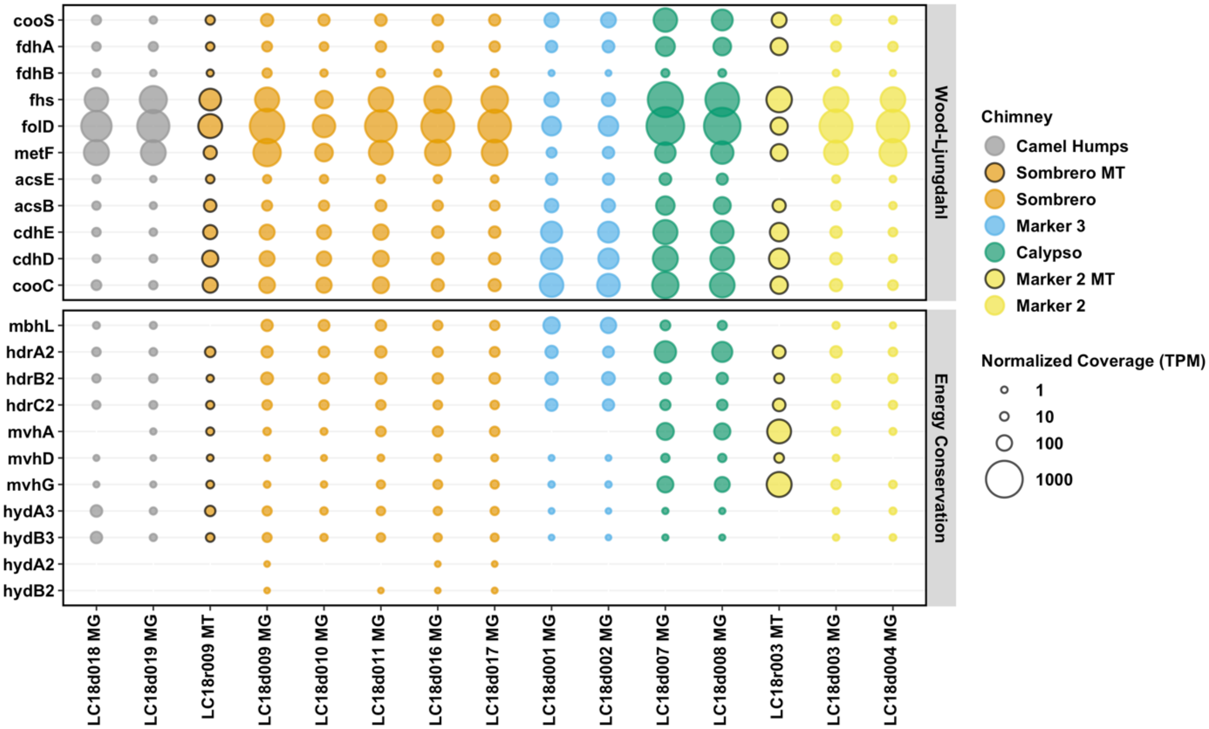

Supplemental Figure S15. Abundance of predicted sequences associated with acetogenesis in Lost City hydrothermal fluid samples. Metagenomic coverage was normalized to predicted protein length and to the size of the metagenome or metatranscriptome library. The final normalized coverage is reported as a proportional unit (transcripts/fragments per million; TPM) suitable for cross-sample comparisons. Bubbles representing coverage in metatranscriptomes (MT), rather than metagenomes (MG), are highlighted with black borders. Genes are defined with KEGG Orthology; see **Supplemental Table S5**.
